# CD8+T cell responsiveness to anti-PD-1 is epigenetically regulated by Suv39h1 in melanomas

**DOI:** 10.1038/s41467-022-31504-z

**Published:** 2022-06-29

**Authors:** Leticia Laura Niborski, Paul Gueguen, Mengliang Ye, Allan Thiolat, Rodrigo Nalio Ramos, Pamela Caudana, Jordan Denizeau, Ludovic Colombeau, Raphaël Rodriguez, Christel Goudot, Jean-Michel Luccarini, Anne Soudé, Bruno Bournique, Pierre Broqua, Luigia Pace, Sylvain Baulande, Christine Sedlik, Jean-Pierre Quivy, Geneviève Almouzni, José L. Cohen, Elina Zueva, Joshua J. Waterfall, Sebastian Amigorena, Eliane Piaggio

**Affiliations:** 1 Institut Curie, PSL Research University, F-75005 Paris, France; 2 INSERM U932, F-75005 Paris, France; 3 Translational Research Department, Institut Curie, F-75005 Paris, France; 4 Université Paris-Est, UMR S955, Université Paris-Est Créteil Val de Marne, Créteil, France; 5 INSERM, U955, Equipe 21, Créteil, France; 6 Institut Curie, PSL Research University, CNRS UMR3666, INSERM U1143, Chemical Biology of Cancer, Equipe Labellisée Ligue contre le Cancer, Paris, France; 7 Inventiva, 50 rue de Dijon, 21121 Daix, France; 8 Institut Curie, Genomics of Excellence (ICGex) Platform, Institut Curie Research Center, Paris, France; 9 Institut Curie, PSL Research University, CNRS, UMR3664, Equipe Labellisée Ligue contre le Cancer, Paris, France; 10 Sorbonne Universités, UPMC University Paris 06, CNRS, UMR3664, F-7005 Paris, France; 11 INSERM U830, F-75005 Paris, France

**Keywords:** Immunotherapy, Cancer immunotherapy, Histone post-translational modifications, Lymphocyte activation

## Abstract

Tumor-infiltrating CD8 + T cells progressively lose functionality and fail to reject tumors. The underlying mechanism and re-programing induced by checkpoint blockers are incompletely understood. We show here that genetic ablation or pharmacological inhibition of histone lysine methyltransferase Suv39h1 delays tumor growth and potentiates tumor rejection by anti-PD-1. In the absence of Suv39h1, anti-PD-1 induces alternative activation pathways allowing survival and differentiation of IFNγ and Granzyme B producing effector cells that express negative checkpoint molecules, but do not reach final exhaustion. Their transcriptional program correlates with that of melanoma patients responding to immune-checkpoint blockade and identifies the emergence of cytolytic-effector tumor-infiltrating lymphocytes as a biomarker of clinical response. Anti-PD-1 favors chromatin opening in loci linked to T-cell activation, memory and pluripotency, but in the absence of Suv39h1, cells acquire accessibility in cytolytic effector loci. Overall, Suv39h1 inhibition enhances anti-tumor immune responses, alone or combined with anti-PD-1, suggesting that Suv39h1 is an “epigenetic checkpoint” for tumor immunity.

## Abstract (web summary)

Understanding CD8 + T cell response to immune checkpoint blockade at the molecular level is important for the design of more efficient cancer immune therapies. Authors show here that the histone lysine methyltransferase Suv39h1 controls the transcriptional programs that determine the functionality of CD8 + T cells and Suv39h1 inhibition may potentiate anti-PD-1 therapy of melanomas.

## Introduction

Surface receptors that inhibit T cell function, or negative checkpoints, play a major role in tumor escape from immune responses. Similar to chronic antigen stimulation during viral infections, T cells inside tumors express high levels of inhibitory receptors and become dysfunctional or exhausted, losing their cytotoxic activity^1,2^. Blocking certain inhibitory receptors with monoclonal antibodies (Ab) has become a major therapeutic tool in cancer patients. Treatment with CTLA-4 or PD-1 blocking Abs can induce potent immune responses against tumors and durable clinical responses. In mouse models, recent studies show critical roles for cytokines (including IL-27) and sequence-specific transcription factors (such as cMAF, TOX, T-bet, and EOMES) in the induction of T cell dysfunction^3,4^. Gene expression programs associated with T cell dysfunction and with re-invigoration of T cell functions after checkpoint blockade have been identified in both humans and mice^5,6^.

These gene expression programs are intimately connected to precise control of chromatin dynamics, which determines the accessibility of genes and regulatory elements to transcription regulators^7^. Schematically, chromatin permissiveness to transcription is associated with chromatin modifiers including so-called writers and erasers, which biochemically modify histone tails, allowing the recruitment of effectors (readers) that modify the structure of the chromatin.

Suv39h1, a histone methyl transferase that promotes trimethylation of lysine 9 of H3 (H3K9me3)^8^, plays critical roles in lymphocyte differentiation. In CD4+ T cells, Suv39h1 is associated with silencing of the IFNγ locus during Th2 differentiation^9^. In the absence of Suv39h1 expression, Th2 cells differentiate, but fail to fully commit to the Th2 fate, and start producing IFNγ in vivo. In CD8+ T cells, Suv39h1 silences the memory/stem cell gene expression program during differentiation of cytotoxic effectors. In the absence of Suv39h1 expression, CD8+ T cell effectors co-express memory genes, and display higher reconstitution potential after adoptive transfer^10^. These results show that Suv39h1-induced heterochromatin dynamics plays a critical role during lymphocyte differentiation, and that chromatin modifiers control specific gene expression programs at precise stages of immune responses. Other epigenetic regulators, including EZH2^11^, DNA methyl-transferases^12^ and histone chaperone CAF-1^13^ are also required to silence stem cell/memory programing during effector differentiation, indicating a general requirement for coordinated changes in chromatin dynamics.

Several recent studies have investigated chromatin accessibility changes during CD8+ T cell differentiation into memory, effector or exhausted phenotypes^6,14,15^. For the latter, transcriptomic and epigenomic changes that occur while T cells become exhausted in the context of chronic viral infection and cancer have been analyzed. A critical question in the field is to understand how blockade of checkpoints, such as anti-PD-1 treatment, reverses the dysfunction of exhausted CD8+ T cells. Recent studies have proposed that reversion of the exhausted phenotype can occur early during the onset of the exhaustion program, and that once this program is fully established it becomes irreversible, and that fully exhausted T cells can no longer respond to PD-1 blockade^15^. Understanding the molecular basis and underlying chromatin dynamics for the establishment of the exhaustion program is a cornerstone with direct consequences for the development of therapeutic strategies in cancer and chronic infections.

In this study, we analyze the role of Suv39h1 in CD8+ T cell exhaustion and re-invigoration by PD-1 blockade in mouse tumors. Using both genetic and pharmacological approaches, we show that Suv39h1 inhibition overcomes tumor resistance to anti-PD-1 treatment. Immune phenotype and single cell RNAseq analysis show that Suv39h1-deficient CD8+ tumor-infiltrating lymphocytes (TIL) display phenotypic characteristics of exhausted cells, including high expression of multiple inhibitory checkpoints. However, the Suv39h1-defective TILs also express a strong IFN-I signature, and respond vigorously to PD-1 blockade, inducing potent tumor rejection and showing broader chromatin accessibility, in particular around genes linked to effector functions, as compared to wild type cells. We conclude that Suv39h1 expression in CD8+ T cells is instrumental for establishing an irreversible, anti-PD-1 resistant exhausted phenotype in tumors. As inhibition of Suv39h1 reverses T cell dysfunction and causes tumor rejection, Suv39h1 represents an actionable “epigenetic checkpoint” for T cell functions in cancer.

## Results

### Suv39h1 deficiency enhances solid tumor rejection in combination with anti-PD-1 Ab

**Fig. 1:**
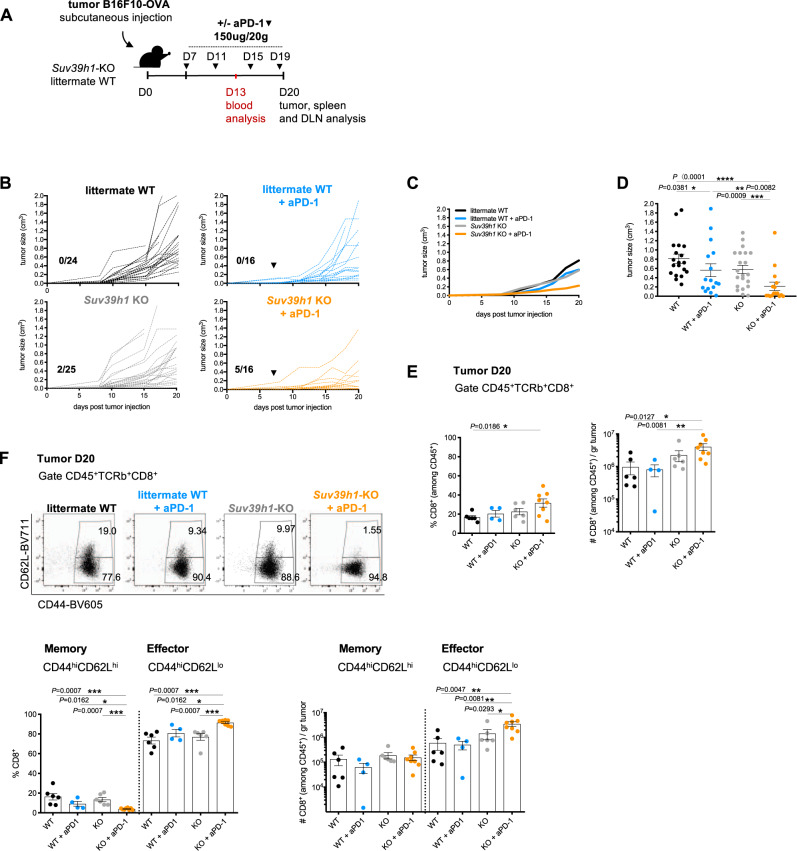
Suv39h1 deficiency inhibits tumor growth in combination with anti-PD-1 Ab. **A** Graphical representation of model system of experimental groups, including littermates WT and *Suv39h1*-KO mice receiving B16F10-OVA melanoma cells followed by PBS or anti-PD-1 Ab. **B** Tumor growth kinetics in B16F10-OVA bearing littermate WT and *Suv39h1*-KO mice receiving PBS or anti-PD-1 Ab. Pooled results from three independent experiments are shown. Numbers refer to rejected tumors out of total mice analyzed. Black arrows indicate time point of initial PBS or anti-PD-1 Ab injection. **C** Tumor growth kinetics. Data is represented as mean. **D** Tumor volumes in cm^3^ (day 20). WT *n* = 19; WT + aPD-1 *n* = 16; KO *n* = 21; KO + aPD-1 *n* = 16. Pooled results from three independent experiments are shown. **E** Frequency (%) and quantification (number) of CD8 + TILs (CD45^+^TCRb^+^CD4^-^). WT *n* = 6; WT + aPD-1 *n* = 4; KO *n* = 6; KO + aPD-1 *n* = 8. A representative experiment out of three is shown. **F** Representative dot plots showing the frequency of memory and effector CD8 + TILs from B16F10-OVA tumors, frequency (%) and quantification (number). WT *n* = 6; WT + aPD-1 *n* = 4; KO *n* = 6; KO + aPD-1 *n* = 8. A representative experiment out of three is shown. *p* values were calculated using two-tailed Mann–Whitney test. *p < 0.05; **p < 0.01; ***p < 0.001; ****p < 0.0001. In all graphs, mean^±^s.e.m. are presented. See also Supplementary Figs. 1 and 2. Source data are provided as a Source Data file.

To interrogate the role of Suv39h1 in T cell responses to cancer, we used a partially anti-PD-1 blockade resistant melanoma model, B16F10-OVA (in which the tumor cells express the surrogate tumor-antigen ovalbumin) grafted in WT or *Suv39h1*-KO mice. Tumor bearing littermate WT and *Suv39h1*-KO mice were randomly assigned to two groups, treated or not with anti-PD-1 monoclonal Ab after establishment of the tumors (Fig. 1A). As expected, anti-PD-1 treatment induces a delay in tumor growth, but not complete rejection in WT mice (Fig. 1B–D). In *Suv39h1*-KO littermates, the growth of the tumor is similarly delayed, with a small number of tumor rejections (2/25), while treatment with anti-PD-1 induces complete tumor rejection in almost one-third of the mice (5/16, Fig. 1B–D). We conclude that Suv39h1 deficiency in the host not only delays tumor growth to levels similar to anti-PD-1 administration, but also synergizes with anti-PD-1 treatment.

The *Suv39h1*-KO mice used here are constitutive knockouts affecting all cell types. To investigate the specific role of Suv39h1-deficient T cells in the observed tumor rejection phenotype, we adoptively transferred in vitro activated control WT and *Suv39h1*-KO OVA-specific T cell receptor (TCR) transgenic OT-1 cells to B6 mice bearing established EL4-OVA tumors (Supplementary Fig. 1A). Mice that received *Suv39h1*-KO OT-1 T cells controlled tumor growth better than mice that received littermate WT OT-1 T cells (Supplementary Fig. 1B), demonstrating that Suv39h1 deficiency in CD8+ T cells augments their anti-tumor potential in the absence of any other Suv39h1-defective cell type.

Also, we generated a new mouse strain, namely the *Suv39h1*-Flox*CD4-Cre, in which mice are KO for *SUV39h1* only in T cells (see the section “Methods” for strain description), and we grafted them with the MCA-101 fibrosarcoma cell line, which is less immunogenic that the B16-F10-OVA (Supplementary Fig. 1C–E). We observed that there is a little tumor growth delay in the *Suv39h1*-KO mice and in the anti-PD-1-treated mice, and that the combination of *Suv39h1* deficiency plus PD-1 blockade induces a higher control of the tumor growth, further demonstrating that the Suv39h1 effect is not restricted to only highly immunogenic tumor types and is T cell intrinsic.

To further understand the implication of Suv39h1-deficient CD8+ T cells in tumor-rejection, we investigated how Suv39h1-defective T cells behave in a Graft vs. Host Disease/Graft vs. Leukemia (GvHD/GVL) model. Irradiated haplotype-matched B6xDBA/2 F1 mice (Hk2bxd; termed B6D2) received a transplant of C57BL/6 (H2-K^b^) bone marrow, with or without Suv39h1-knockout (KO) or wild-type littermate (WT) T cells, before being challenged with P815-GFP murine mastocytoma cells (H2-K^d^) (Supplementary Fig. 1F). Both *Suv39h1*-KO and littermate WT T cells induced a modest increase in survival (10-30%), as compared to the control group that did not receive T cells (Supplementary Fig. 1G). To determine if the mice died due to tumor or to GvHD development, tumor growth (Supplementary Fig. 1H, I) and GvHD severity (Supplementary Fig. 1J, K) were measured. While mice injected with WT T cells showed a high tumor incidence (more than 60% had detectable tumor cells) and developed mild GvHD, the mice injected with *Suv39h1*-KO T cells showed instead a lower tumor incidence (20%) and developed more severe GvHD. Indeed, while mice injected with WT T cells died from tumor development, the ones injected with Suv39h1-deficient T cells died from GvHD (78%) (Supplementary Fig. 1L). Therefore, in this adoptive T cell transfer model Suv39h1-deficient T cells develop into much more potent effectors (that reject the tumor and attack the host) than their Suv39h1-proficient counterparts.

These two latter experiments (Supplementary Fig. 1) suggest that the increased tumor control observed in Suv39h1-defective mice (Fig. 1A–D) could be attributed, at least partially, to an increased anti-tumor activity of *Suv39h1*-KO CD8+ T cells. Along these lines, the percentages of tumor-infiltrating myeloid cells (CD11b+ cells, cDC1, and cDC2 cells), CD4+ T cells, and NK and were similar in untreated WT and *Suv39h1*-KO animals, and were not significantly modified by the anti-PD-1 treatment (Supplementary Fig. 2A); while the percentage and absolute number of CD8+ TILs were increased in the *Suv39h1*-KO mice as compared to WT littermates, and were expanded further after treatment with anti-PD-1 Ab (Fig. 1E). The increase in CD8+ T cells was restricted to the tumor, as no significant changes were observed in the tumor-draining lymph nodes (DLN) (Supplementary Fig. 2B). These results highlight the link between increased CD8+ T-cell infiltration and tumor rejection in anti-PD-1 Ab treated *Suv39h1*-KO mice.

It has been previously described that Suv39h1 is a critical regulator of the transition of CD8+ T cells between the memory and effector differentiation states^10^. Thus, we analyzed the proportions of naïve (CD44loCD62Lhi), central memory (CD44hiCD62Lhi) and effector (CD44hiCD62Llo) CD8+ T cell subsets in blood (during tumor growth progression, at day 13), and in spleen, DLNs, and tumor (day 20). We observed that in general, compared to WT mice, *Suv39h1*-KO littermates treated or not with anti-PD-1, showed lower proportions of naïve CD8+ T cells and higher proportions of effector and central memory subsets, in the three locations (Supplementary Fig. 2C–E). In contrast, in the tumor, virtually no naïve CD8+ T cells were detected, and the main effect was observed in anti-PD-1 treated *Suv39h1*-KO mice, in which the proportion of memory cells (CD44hiCD62Lhi) was significantly lower, while both the proportion and the absolute number of effector CD8+ T cells were significantly higher (Fig. 1F). Therefore, the absence of Suv39h1 expression not only leads to higher CD8+ T-cell tumor-infiltration upon anti-PD-1 treatment, but also a change in the phenotype of the infiltrating cells, away from memory and towards a more pronounced effector phenotype.

### *Suv39h1*-KO CD8+ TILs display enhanced functional capacity

**Fig. 2:**
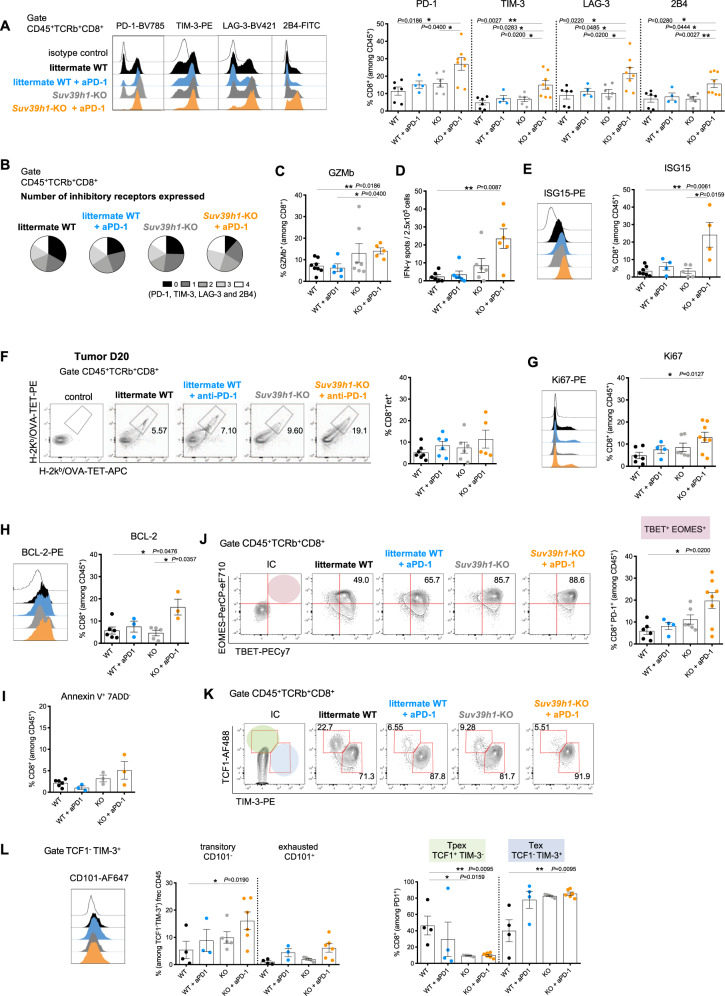
PD-1 blockade in *Suv39h1*-KO CD8+ TILs modifies their exhaustion program and enhances their effector capacity. **A** Representative histogram and frequency (%) of inhibitory receptors (PD-1^+^, TIM-3^+^, LAG-3^+^ and 2B4^+^) on CD8 + TILs from B16F10-OVA tumors. WT *n* = 6; WT + aPD-1 *n* = 4; KO *n* = 6; KO + aPD-1 *n* = 8. **B** Pie chart of co-expression (PD-1^+^, TIM-3^+^, LAG-3^+^, and 2B4^+^) on CD8+ TILs from B16F10-OVA tumors. **C** Representative histogram and frequency (%) of GZMb^+^ cells among CD8+ TILs. Cells were re-stimulated in vitro with PMA and ionomycin for 4 h. (GZMb: Granzyme b). WT *n* = 8; WT + aPD-1 *n* = 5; KO *n* = 7; KO + aPD-1 *n* = 5. **D** Blood cells from mice were re-stimulated ex vivo with OVA-I_SIINFEKL_ peptide. The number of OVA-specific T cells producing IFNγ per 2.5 × 10^5^ blood cells was determined by ELISPOT analysis. WT *n* = 6; WT + aPD-1 *n* = 6; KO *n* = 6; KO + aPD-1 *n* = 6. **E** Representative histogram and frequency (%) of ISG15^+^ among CD8 + TILs from B16F10-OVA tumors. WT *n* = 7; WT + aPD-1 *n* = 4; KO *n* = 5; KO + aPD-1 *n* = 4. **F** Representative dot plots and frequency (%) of OVA-specific TILS from B16F10-OVA tumors as detected using Ova-SIINFEKL/Kb tetramers coupled to two different fluorochromes. WT *n* = 7; WT + aPD-1 *n* = 6; KO *n* = 6; KO + aPD-1 *n* = 5. **G** Representative histogram and frequency (%) of Ki67^+^ among CD8+TILs from B16F10-OVA tumors. WT *n* = 6; WT + aPD-1 *n* = 4; KO *n* = 6; KO + aPD-1 *n* = 8. **H** Representative histogram and frequency (%) of BCL-2^+^ among CD8+ TILs from B16F10-OVA tumors. WT *n* = 6; WT + aPD-1 *n* = 3; KO *n* = 5; KO + aPD-1 *n* = 3. **J** Representative contour plots and frequency (%) of TBET^+^EOMES^+^ among CD8 + PD-1^+^ TILs from B16F10-OVA tumors. WT *n* = 6; WT + aPD-1 *n* = 4; KO *n* = 6; KO + aPD-1 *n* = 8. **K** Representative contour plots and frequency (%) of progenitor exhausted (TCF1^+^TIM-3^−^) and late exhausted (TCF1^−^TIM-3^+^) among CD8^+^ PD-1^+^ TILs from B16F10-OVA tumors. WT *n* = 4; WT + aPD-1 *n* = 4; KO *n* = 5; KO + aPD-1 *n* = 6. **I** Frequency (%) of Annexin V^+^ 7ADD^−^ among CD8 + TILs from B16F10-OVA tumors. WT *n* = 6; WT + aPD-1 *n* = 3; KO *n* = 3; KO + aPD-1 *n* = 3. **L** Representative histogram and frequency (%) of transitory (CD101^-^) and exhausted (CD101^+^) among CD8^+^ PD-1^+^ TCF1^-^TIM-3^+^ TILs from B16F10-OVA tumors. WT *n* = 4; WT + aPD-1 *n* = 3; KO *n* = 5; KO + aPD-1 *n* = 6. A representative experiment out of two is shown. *p* values were calculated using two-tailed Mann–Whitney test. *p < 0.05; **p < 0.01; ***p < 0.001; ****p < 0.0001. In all graphs, mean^±^s.e.m. are presented. See also Supplementary Fig. 3. Source data are provided as a Source Data file.

A vast body of literature shows that, upon tumor invasion, CD8+ T cells differentiate into an exhausted and unresponsive state in late-stage tumors^15^. Because tumors are rejected more efficiently in Suv39h1-defective mice treated or not with anti-PD-1, we hypothesized that the absence of Suv39h1 could delay or prevent exhaustion programs and together with checkpoint blockade could overcome dysfunctional states. To further investigate if Suv39h1-defective TILs are exhausted, we first analyzed the expression of negative-immune checkpoints, which represent a hallmark of exhaustion^16^. In *Suv39h1*-KO mice, treatment with anti-PD-1 caused a marked increase in the proportion of TILs expressing PD-1, TIM-3, LAG-3, and 2B4 (Fig. 2A) and of all combinations of these receptors (Fig. 2B), as well as other surface molecules associated with exhaustion, such as CD39, CD38 and CD101 (Supplementary Fig. 3A), suggesting more advanced differentiation^15,17,18^. This increase was not observed in WT littermates treated in the same way.

To further study the effect of anti-PD-1 treatment on the activation state of Suv39h1-defective CD8+ TILs, we analyzed their effector functions, proliferation and survival, which are impaired in exhausted T cells^19,20^. We observed that upon ex vivo stimulation CD8+ TILs from *Suv39h1*-KO mice expressed the highest levels of Granzyme B (GZMb), a marker of cytotoxic function (Fig. 2C), readily produced IFNγ, as detected by ELISPOT (Fig. 2D), and responded to IFN, as indicated by the high expression of ISG15 (a hallmark gene of IFN-response) (Fig. 2E).

To quantify the OVA-specific CD8+ T cell response, TILs obtained from B16-OVA tumor were stained with OVA-specific tetramers (Fig. 2F). We observed that the OVA-specific response concerns a minor fraction of the TIL population, which does not change significantly by Suv39h1 status or anti-PD-1 alone treatment (mean % of CD8 + T cells ± SEM: 5.3 ± 1.2% for WT, 85 ± 2.0% for WT + anti-PD-1; 7.5 ± 2.5 for Suv39h1-KO). The proportion of OVA-specific CD8+ T cells, however, increases in a subgroup of anti-PD-1-treated Suv39h1 KO mice (11,5% ± 4,2), following a similar trend to the ELISPOT responses.

Furthermore, around 13% of CD8+ TILs from PD-1-treated *Suv39h1*-KO mice expressed higher levels of Ki67 (a marker of cycling cells, Fig. 2G). Nevertheless, increased cycling was not associated with augmented apoptosis, as suggested by the increased expression of BCL-2 (an anti-apoptotic molecule) (Fig. 2H) and small proportions of annexinV/7ADD+ cells (Fig. 2I), suggesting conserved viability. To further understand this intriguing observation, we analyzed the expression of T-box transcription factors EOMES and TBET, both crucial markers in effector and memory functions in T cells^19,21,22^, as well as the HMG-box transcription factor TOX, primary regulator of exhaustion epigenetic program^22–26^. We observed similar levels of TOX in CD8+ TILs from all mice conditions, and increased levels of EOMES in anti-PD-1 treated *Suv39h1*-KO mice (Supplementary Fig. 3B). As cells get activated and enter into an exhaustion program, they upregulate the expression of Eomes; however, co-expression of EOMES with TBET has been associated with a rescue from a terminally exhausted state, as PD-1+EOMES+ TBET+ cells retain effector functions^4,19,27^. We observed that CD8+ PD-1+ TILs co-expressing EOMES and TBET where enriched in anti-PD-1 treated *Suv39h1*-KO mice (Fig. 2J). These results indicate the ability of *Suv39h1*-deficient CD8+ TILs to rewire the transcriptional control of TBET and EOMES after PD-1 blockade, independently of TOX expression. Thus, although Suv39h1-defective CD8+ TILs display features of exhausted cells (e.g. express high levels of negative checkpoints and EOMES), they are found in increased numbers (Fig. 1E), produce IFNγ and GZMb and importantly are not apoptotic, indicating enhanced poly-functionality and survival.

It has been recently described that anti-PD-1 treatment may work not only by reinvigorating exhausted TILs, but also by inducing the proliferation of progenitor exhausted cells with memory/stem cell-like characteristics, as well as their differentiation into cytotoxic and short-lived, terminally exhausted cells resistant to anti-PD-1 therapy^28,29^. We quantified progenitor and terminally exhausted T cells, identified as TCF1+ TIM3− or TCF1-TIM3+ cells, respectively, among memory CD8+ PD-1+ TILs^28^. We observed that in the tumors of Suv39h1-KO mice, the proportion of stem-like progenitor exhausted CD8+ T cells was lower than in their WT littermates, and the percentage of terminally exhausted T cells was significantly higher in anti-PD-1 treated Suv39h1-KO CD8+ T cells, compared with WT mice (Fig. 2K). These results suggest that in absence of Suv39h1 activity, CD8+ TILs differentiate from stem cell-like, progenitor-exhausted T cells to a more effector, terminal exhausted phenotype. Similar results were obtained when using the cell surface marker SLAMF6 instead of the transcription factor TCF-1^28^ to distinguish precursor and later effector populations (Supplementary Fig. 3C).

Recent studies in chronic viral infection demonstrated that the expression of the type I transmembrane glycoprotein CD101 subdivide the terminally exhausted population into two subgroups^16^. Stem cell-like progenitor exhausted TCF1+ CD8+ T cells first differentiate into a transitory CD101-TIM3+ state marked by an effector-like profile displaying chemokine receptor CX3CR1, cytokines and GZMb expression, contribute to viral control and eventually progress to a terminally differentiated and dysfunctional CD101+ TIM3+ state. Notably, Hudson et al.^17^ observed that PD-1 blockade increases the transitory cytolytic effector-like CD101-TIM3+ cells compared to stem-like CD8+ T cells. In our tumor model, we observed that anti-PD-1 treatment induced a subtle increase in the percentages of terminally exhausted CD101+ TIM3+ cells in both WT and Suv39h1KO CD8+ TILs. The increase of transitory CD101-TIM3+ cells, in contrast, was stronger in Suv39h1-defective mice relative to the control littermates (Fig. 2L). These results indicate that in the absence of SUV39H1, anti-PD-1 treatment increases the proliferative/effector T cell differentiation by enhancing the conversion to transitory exhausted CD101-TIM3+ T cells. To understand the relative contribution of OVA-Tet+ cells to the observed phenotype of the global CD8+ population, we compared the expression of some inhibitory receptors in OVA-Tet+ vs. OVA-Tet- CD8+ TILS (Supplementary Fig. 3D). We observed that Tet+ cells express similar levels of PD-1, LAG-3, and CD39 independent of the experimental condition (almost 100% are positive for PD1 and CD39, and around 80% for LAG-3). These results indicate that changes observed in bulk CD8+ TILS cannot uniquely be attributed to an outgrowth of OVA-specific cells, or to a change in their phenotype, but that changes are mainly contributed by OVA-Tet- cells.

### scRNAseq analysis of tumor-infiltrating CD8+ T cells

**Fig. 3:**
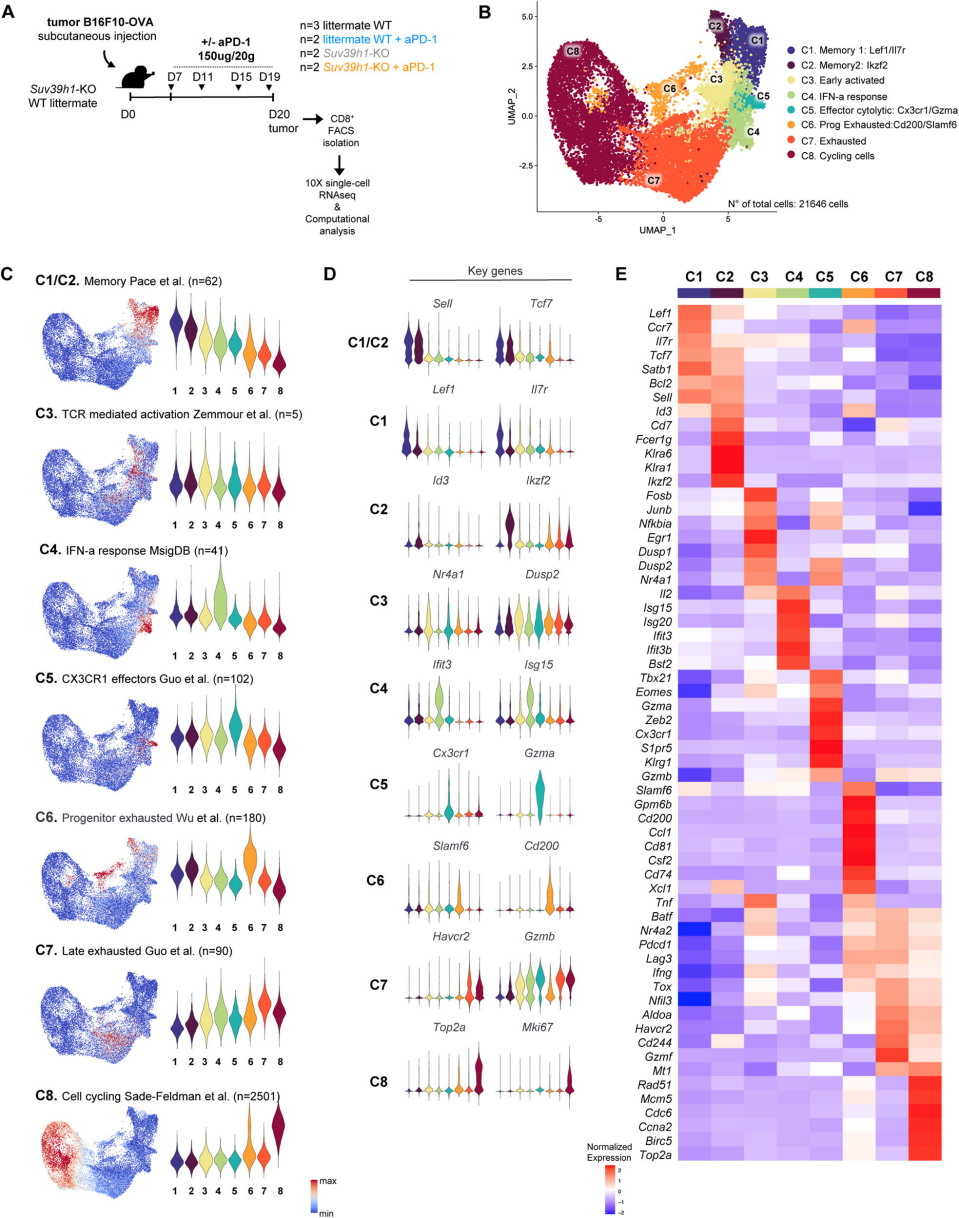
Identification of multiple CD8+ TIL populations by single cell RNA sequencing. **A** Scheme of the overall study design. Single–cell RNA sequencing was applied to CD8+ TILs isolated from littermate WT and *Suv39h1*-KO mice receiving B16F10-OVA melanoma cells followed by PBS or anti-PD-1 Ab. **B** UMAP plot with clusters differentiated by colour demonstrating eight distinct CD8+ TILs clusters based on gene expression differences for 21646 passing quality control cells. Each dot corresponds to one single cell. **C** Feature plots and violin plots showing distribution of signature scores indicated by cluster. **D** Violin plots showing expression distribution of representative genes for each cluster. **E** Heatmap showing *Z* scores for the average expression of selected genes within the eight clusters. See also Supplementary Fig. 4. Source data are provided as a Source Data file.

To further characterize the impact of Suv39h1 on TIL programing, we performed single cell RNA sequencing (scRNAseq) of CD8+ TILs isolated from B16F10-OVA tumors of *Suv39h1*-KO and littermate WT mice treated or not with anti-PD-1 (Fig. 3A). In total, 21646 cells (1524–5953 cells per sample) were merged, and 8 clusters were defined, which are visualized using UMAP in Fig. 3B (see Supplementary Fig. 4 and Methods section for bioinformatics pipeline details). Figure 3 shows the signatures used to define cluster identities (Fig. 3C), as well as key exemplifying genes (violin plots, Fig. 3D; and heatmap, Fig. 3E). Supplementary Fig. 4D, E shows additional information used to define each cluster. In more detail, cluster 1 and 2 were composed of cells bearing high memory signatures: “C1-memory 1” characterized by high expression of *Lef1* and *Il7r*; and “C2-memory 2” by *Id3*, *Ikzf2*/*Helios* and killer cell lectin-receptors (*Klra1* and *Klra6*); cluster 3, “C3-early activated” included cells with a signature of recent TCR-engagement, as featured by the expression of *Nr4a1* and *Dusp2*; cluster 4, “C4-IFN-a response” expressed an IFN-a-induced gene signature; cluster 5, “C5-effector cytolytic”, shared similarities with the previously described short-lived effector T cell cytolytic cluster required to control chronic viral infection containing the chemokine receptor Cx3cr1, genes associated with cytotoxicity (*Gzma*, *Klrg1* and *Gzmb*) and the transcription factors *Zeb2* and *Klf3*^18,30,31^; cluster 6, “C6-progenitor exhausted” was composed of cells highly expressing *Ccr7*, *Id3*, *Slamf6*, *Cd200* and *Cd74* indicative of progenitor stem cell-like exhausted cells; cluster 7, “C7-terminally exhausted” showed a late exhausted signature and highly expressed the pathognomonic genes *Havcr2*, *Gzmb*, and *Mt1* (encoding metallothionein-1), which has been described to promote T-cell dysfunction during cancer^32^; and cluster 8, “C8-cycling” expressed a cell cycle signature and the characteristic active cell cycle phase genes *Top2a* and *Mki67*. Altogether, these results identify a high heterogeneity among CD8+ TIL populations, likely reflecting a broad functional specialization.

### Suv39h1-deficient TILs show an IFN-a response signature and a higher cytolytic potential upon PD-1 blockade

**Fig. 4:**
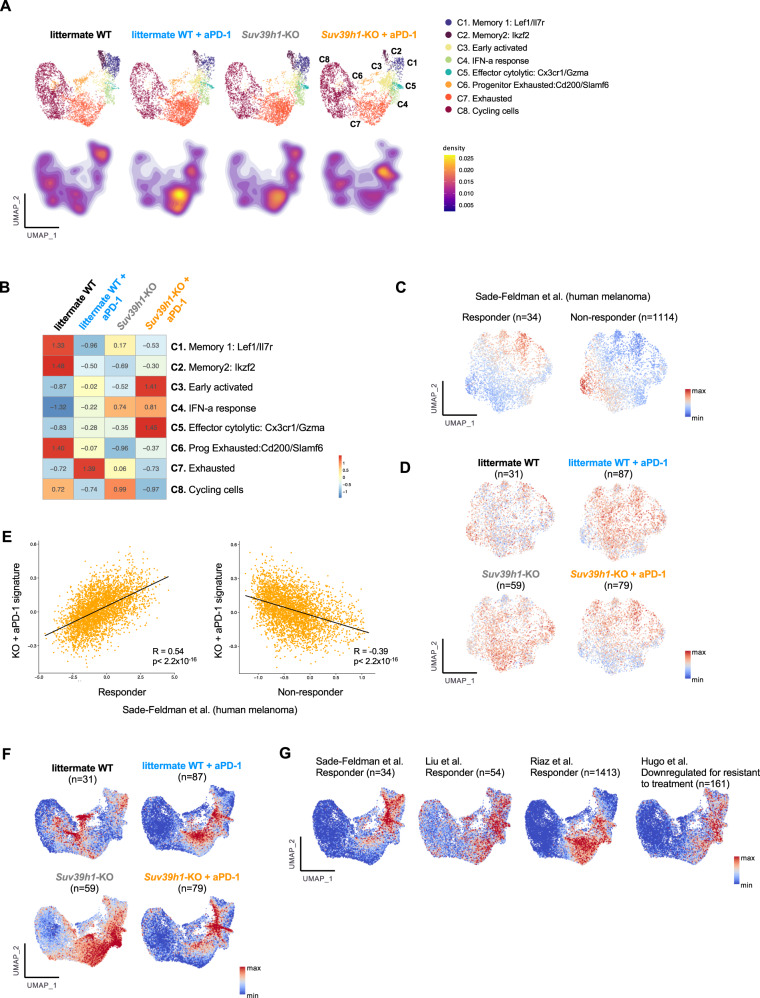
PD-1 blockade in *Suv39h1*-KO CD8+TILs induces a transcriptomic effector phenotype also found in responder melanoma patients. **A** UMAP plot of 4097 cells for each of the four analyzed conditions after downsampling (on the top) and the corresponding density plots (on the bottom). **B** Heatmap of proportion and numeric value of the downsampled 4097 cells for each of the four conditions within the eight clusters. **C** Feature plots of human melanoma CD8+ TILs showing responder and not responder signature score from scRNAseq published dataset^32^. **D** Feature plots of human melanoma CD8+ TILs showing distribution of signature scores from littermate WT and Suv39h1-KO treated or not with anti-PD-1 dataset conditions. **E** Correlation between transcriptomic signatures from *Suv39h1*-KO mice treated with anti-PD-1 and the responder and not responder melanoma patients^32^ calculated with Pearson correlation coefficient. **F** Feature plots of murine CD8+ TILs (Fig. 3B) highlighting distribution of signature scores of littermate WT and *Suv39h1*-KO treated or not with anti-PD-1 dataset conditions. **G** Enrichment of the transcriptomic signature of genes upregulated in published melanoma ICB responders projected in murine CD8+ TILs UMAP (Fig. 3B). See also Supplementary Fig. 5. Source data are provided as a Source Data file.

To further investigate how treatment with anti-PD-1 Ab affects CD8+ TILs in WT and *Suv39h1*-KO mice, we then explored the transcriptomic differences of the clusters between conditions. All clusters were present in each of the four analyzed conditions, but in different proportions, and key genes characterizing cluster identity were not overtly modified by the mouse condition and/treatment, validating the chosen cluster definition to subsequently measure changes in cell proportions in each cluster upon treatment (Supplementary Fig. 4F). Thus, we first performed downsampling, so as to analyze the same amount of cells in each condition (randomly sampling 4097 cells from each condition to match the smallest one, *n* = 16,388 total cells after downsampling), and then quantified the changes in the proportions of cells belonging to each cluster from treated and untreated, littermate WT and *Suv39h1*-KO mice. As shown in Fig. 4A, B, CD8+ TILs from littermate WT untreated mice contained the highest proportions of memory and progenitor exhausted cells compared to all the other conditions. Upon anti-PD-1 treatment, these populations seemed to evolve into an exhausted state. Distinctively, in untreated conditions *Suv39h1*-defective TILs were characterized by higher proportions of “IFN-responding” and “exhausted” cells than WT cells. Upon anti-PD-1 treatment, *Suv39h1*-KO CD8+ T cells comprised the highest frequencies of early activated, effector cytolytic and IFN-responsive cells, but at odds with the FACS data (Fig. 2F) that showed increased proportions of Ki67-positive cells in *Suv39h1*-KO animals receiving anti-PD-1, the frequencies of the cycling cell cluster defined by scRNAseq (which regroups cells in different phases of the cell cycle, see (Supplementary Fig. 4B, C) did not reflect these changes. Overall, these results suggest that along the activation/differentiation program of anti-tumor T cells, Suv39h1-dependent silencing of the IFN-I response and of pathways of the exhaustion program, imposes an epigenetic barrier that prevents re-programing of TILs towards a terminally exhausted program by anti-PD-1. Moreover, our data indicate that in the absence of Suv39h1, CD8+ TILs are poised to more effectively respond to TCR-activation and to become highly cytolytic upon PD-1-blockade, underlying the increased tumor rejection compared to WT mice.

### The anti-PD-1 response signature of Suv39h1-deficient CD8+ T cells discriminates response to anti-PD-1 in melanoma patients

We then asked whether the signature of *Suv39h1*-KO CD8+ T cells that increase after anti-PD-1 in the mice is relevant to human cancer. To address this question, we first interrogated whether our data correlated with available published signatures from scRNAseq of CD8+ T cells from melanoma patients responding or not to immune checkpoint blockade^33^. We first confirmed that the “responder” and “non-responder” signatures from the original paper correspond to distinct subpopulations of cells in the human dataset (Supplementary Fig. 5A, B and Fig. 4C). To compare mice and human anti-PD-1 responses, we computed ortholog-matched signatures of each experimental group (littermate WT, littermate WT+ anti-PD-1, KO, and KO+ anti-PD-1) and quantified them on the reprocessed human melanoma cohort UMAP (Fig. 4D). We observed that the KO+ anti-PD-1 signature best matched with the “responder” signature from the original study, and that they were strongly positively correlated (*R* = 0,54, *p* < 2.2e−16) (Fig. 4E). Interestingly, the KO+ anti-PD-1 signature was also anti-correlated with the “non-responder” signature (*R* = −0.39, *p* < 2.2e−16). Finally, we also noticed an enrichment of the KO + anti-PD-1 signature coming from our mice data in responding patients (*p* = 4.8 × 10^−12^) (Supplementary Fig. 5C), suggesting that our KO+ anti-PD-1 signature could be a potential biomarker of response to immune checkpoint blockage (ICB) therapy.

We next used our mouse scRNAseq data to identify the specific subtype of CD8+ T cell response induced by the treatment in the responder patients. We first plotted signatures of the different conditions (littermate WT, littermate WT+ anti-PD-1, KO, KO+ anti-PD-1) (Fig. 4F) and compared them with four previously published melanoma ICB response signatures^33–36^ (Fig. 4G). While each of these four previous studies differ with regards to detailed patient clinical characteristics, RNAseq profiling techniques and analytical approaches, we observed that across the four studies, the human signatures of response to immunotherapy were consistently associated to the cytolytic effector, TCR activation and IFN type I clusters, which are also those upregulated in the mouse KO+ anti-PD-1 CD8+ T cells, and to a lesser degree to an increase of a fraction of “terminally exhausted” cells.

Overall, the transcriptional program induced by anti-PD-1 in the absence of Suv39h1 activity is associated with human T cells responding to PD-1 blockade and clearly links the emergence of effector CD8+ T cell subpopulations to the effectiveness of immune checkpoint blockade.

### Suv39h1-deficiency enhances chromatin accessibility induced by anti-PD-1

**Fig. 5:**
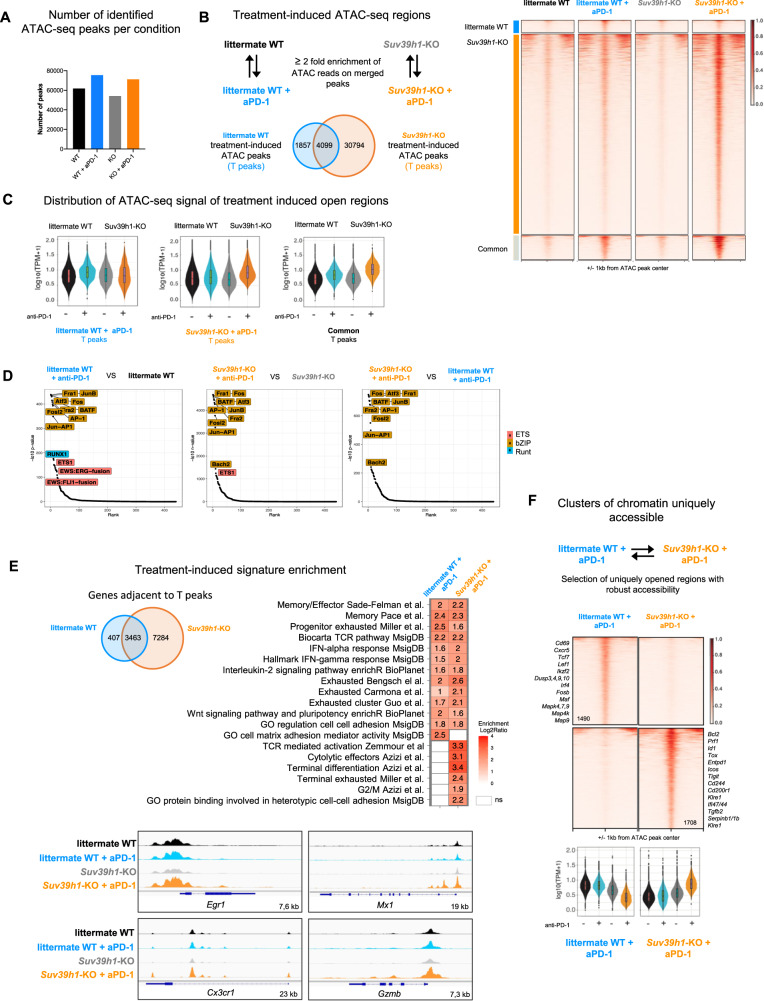
Suv39h1-deficiency enhances anti-PD-1-induced chromatin opening at TCR-, cytolytic-, and IFNa-responsive sites. **A** Number of chromatin accessible regions in CD8+ TILs of littermate WT and *Suv39h1*-KO mice treated or not with anti-PD-1. **B** ATAC-seq regions in littermate WT and *Suv39h1*-KO modulated by anti-PD-1 treatment. Venn diagram showing anti-PD-1 treatment modulated peaks in littermate WT, *Suv39h1*-KO, or both (overlap) (middle). Chromatin accessibility heat map grouped by modulated peaks by anti-PD-1 treatment in CD8^+^ TILs of littermate WT, *Suv39h1*-KO, or both (bottom). **C** Violin plots showing distribution of anti-PD-1 induced ATAC-seq signals in CD8+ TILs littermate WT and *Suv39h1*-KO CD8+ TILs. Central line represents median, box represents quartiles, whiskers represent minimal to maximal data value. **D** Motif enrichment analysis showing top transcription factor motifs enriched in peaks differentially accessible in CD8+ TILs of the described group comparisons. **E** Venn diagram showing number of genes adjacent to anti-PD-1 induced ATAC-seq peaks in littermate WT and *Suv39h1*-KO CD8+ TILs (top). Selected signatures enriched in anti-PD-1 induced adjacent peaks open in littermate WT and *Suv39h1*-KO CD8+ TILs determined through hypergeometric test alternative greater (middle). Representative ATAC-seq tracks showing accessibility peaks across the loci of *Egr1*, *Mx1*, *Cx3cr1* and *Gzmb* for littermate WT and *Suv39h1*-KO treated or not with anti-PD-1 CD8^+^ TILs (bottom). **F** Chromatin accessibility heat map grouped by unique differentially accessibility regions in CD8^+^ TILs of littermate WT+ anti-PD-1 and *Suv39h1*-KO+ anti-PD-1 (top). Violin plots showing distribution of unique anti-PD-1 signals in CD8+ TILs of littermate WT+ anti-PD-1 and *Suv39h1*-KO+ anti-PD-1. Central line represent median, box represent quartiles, whiskers represent minimal to maximal data value. All ATAC-seq data are representative of two biologically independent pooled samples. Source data are provided as a Source Data file.

To investigate the effect of anti-PD-1 tratment on chromatin accessibility in WT and *Suv39h1*-KO T cells, we next used *Assay for Transposase-Accessible Chromatin with high throughput sequencing* (ATAC-seq). CD8+ TILs were FACS-sorted from tumors growing in WT and *Suv39h1*-KO mice, treated or not with anti-PD-1. Accessible regions were first identified by calling ATAC-peaks for each condition. Tens of thousands of peaks were detected in each sample, with numbers systematically higher for anti-PD-1-treated cells, both in WT and *Suv39h1*-KO CD8+ T cells, relative to untreated samples (Fig. 5A). To get quantitative insight into changes caused by anti-PD-1 treatment, we first compared treated samples directly to their untreated genotype-matched counterparts. An ATAC peak (identified in either condition) was considered modulated if it harbored twice or more sequencing reads (normalized for sequencing depth) as compared to the corresponding counterpart (Fig. 5B, left panel).

No regions emerged as enriched in anti-PD-1 untreated relative to treated samples. On the contrary, anti-PD-1 treatment is assymetrically associated with increased chromatin accessibility, in both WT and *Suv39h1*-KO backgrounds (Fig. 5B, left panel). In WT cells, almost 6000 regions at least double their ATAC-signal, as compared to their untreated counterparts. Reminiscent of previous studies on the role of DNA methylation and DNMT3A in anti-PD1 responses^37^, *Suv39h1*-KO cells show an even more dramatic treatment effect, with 35000 genomic regions gaining accessibility (almost half of all identified ATAC peaks in KO cells). In WT CD8+ TILs, the majority (68%, 4009/5866) of treatment-induced regions (T-peaks) are shared with *Suv39h1*-KO T-peaks (Fig. 5B, left panel). ATAC signal density plots generated for 20% of the most variable T-peaks (Fig. 5B, right panel) reveal well-defined clusters: (i) *Suv39h1*-KO-specific, (ii) WT-specific, and (iii) common to both genotypes. To cross-compare accessibilities of identified treatment-specific peaks in all four conditions, we plotted the distribution of ATAC signal in peaks for each cluster separately. As expected, average signals in WT or *Suv39h1*-KO treatment-specific peaks are higher, as compared to the corresponding untreated counterparts (Fig. 5C). Importantly, a stronger signal in *Suv39h1*-KO cells on shared T-peaks suggests that they are more accessible in a higher proportion of cells, as compared to WT. These results indicate that anti-PD-1 treatment promotes chromatin accessibility, and that this effect is more pronounced in the absence of Suv39h1. Alternatively, anti-PD-1 treatment could also be expanding cells that possess open chromatin regions. Previous studies have shown that while T cell chromatin changes do occur upon anti-PD-1 treatment, they do not correspond to full reprogramming into memory phenotypes^6,14^. Consistent with these results, motif enrichment analyses show that anti-PD-1 treatment is strongly associated with increased chromatin accessibility at AP-1 binding-motifs sites, in both WT and *Suv39h1*-KO backgrounds, consistent with increased TCR signaling activity (Fig. 5D). This enrichment was stronger in the KO background, compared to WT cells (compare vertical axes scales), which is also reflected when comparing the differentially accessible sites between WT and KO anti-PD1 treated samples.

To reveal biological signatures associated with anti-PD-1 treatment, we next assigned treatment-specific peaks to their nearest genes. We observed that the majority of genes assigned to treatment-specific peaks in WT cells are shared with *Suv39h1*-KO (Fig. 5E, left panel), but conversely, the majority of treatment-specific peaks in *Suv39h1*-KO cells are not differentially accessible in WT cells. Signature enrichment analysis showed that anti-PD-1 treatment leads to an increase in chromatin accessibility in genomic loci linked to T cell memory, pluripotency and activation (TCR pathway, IL-2 pathway, IFNγ-response, and exhaustion), in both WT and *Suv39h1*-KO CD8+ T cells (Fig. 5E, right panel). The progenitor signature, however, is more enriched in WT, while the IFNγ- and IFN-I signatures are more enriched in KO cells. Moreover, multiple effector signatures (cytolytic, terminally differentiated, as well as cell cycle) are uniquely associated with treatment-specific peaks in *Suv39h1*-defective cells. Figure 5E (lower panel), shows loci of representative TCR-mediated activation gene *Egr1*, effector genes (*Cx3cr1* and *Gzmb*) and IFN-stimulated gene *Mx1*, that become more accessible in *Suv39h1*-KO cells after anti-PD-1 treatment, as compared to control conditions. These results suggest that, altogether, in anti-PD-1 treated cells chromatin accessibility is increased at genomic loci linked to T cell activation, memory and pluripotency, but in a *Suv39h1*-KO context cells acquire preferential accessibility at functional IFN-related and cytolytic effector loci as compared to WT cells.

To investigate the differences between WT and *Suv39h1*-KO populations after anti-PD-1 treatment, we next compared chromatin opening in the two populations. We only retained ATAC regions harboring twice or more reads and, importantly, called as peaks uniquely open in one, but not the other population after the treatment (Fig. 5F, upper panel). Of these peaks, around 1500 in WT and 1700 in KO are identified as condition-specific (see density plots in Fig. 5F with selected adjacent genes). Peaks uniquely accessible in treated WT cells (when compared to treated KO cells), are equally accessible in untreated WT population, (see ATAC signal distribution in Fig. 5F, lower panel). Only 3% of the peaks are modulated by the treatment, suggesting that these loci are stably opened regions in WT TILs. Peaks specific to treated *Suv39h1*-KO cells (compared to treated WT cells), are also accessible in untreated KO samples, although over half of them (58%) respond to the treatment with a strong increase in accessibility (Fig. 5F, lower panel). Therefore, in the absence of Suv39h1, a series of chromatin regions, mostly treatment-sensitive, fail to remain closed. As ATAC-regions are typically associated with active or poised regulatory elements^38^, these results collectively suggest that Suv39h1 may contribute to the regulation of chromatin opening at cis-regulatory elements that support differentiation of T cells into functional cytolytic effectors upon re-activation.

### Pharmacological inhibition of Suv39h1 phenocopies the genetic defect

**Fig. 6:**
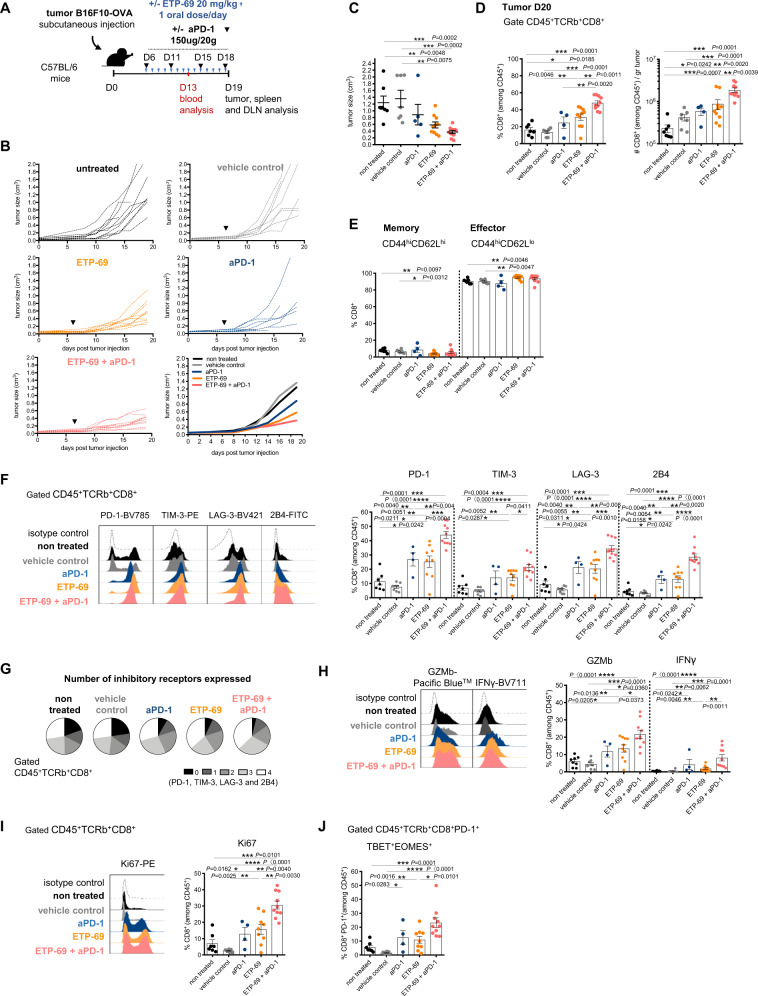
Pharmacological inhibition of Suv39h1 potentiates tumor rejection by anti-PD-1 Ab. **A** Graphical representation of model system of experimental groups, including C57BL/6 mice receiving B16F10-OVA melanoma cells followed by ETP-69 oral treatment, PBS or anti-PD-1 Ab injection. **B** Tumor growth kinetics represented as means of one representative experiment out of 2; with *n* = 8 to 10 mice per group. Black arrows indicate time of initial ETP-69 or vehicle control dose administration and anti-PD-1 Ab injection. non treated *n* = 10, vehicle control *n* = 8; aPD1 *n* = 8; ETP-69 *n* = 10; ETP-69 + aPD-1 *n* = 10. **C** Tumor volumes in cm^3^ (day 19). non treated *n* = 7, vehicle control *n* = 7; aPD1 *n* = 5; ETP-69 *n* = 10; ETP-69 + aPD-1 *n* = 10. **D** Frequency (%) and quantification (number) of CD8 + TILs (CD45^+^TCRb^+^CD4^-^). nonreated *n* = 7, vehicle control *n* = 7; aPD1 *n* = 4; ETP-69 *n* = 10; ETP-69 + aPD-1 *n* = 10. **E** Frequency (%) of memory and effector CD8+ TILs from B16F10-OVA tumors. nontreated *n* = 7, vehicle control *n* = 6; aPD1 *n* = 4; ETP-69 *n* = 10; ETP-69 + aPD-1 *n* = 10. **F** Representative histogram and frequency (%) of inhibitory receptors (PD-1^+^, TIM-3^+^, LAG-3^+^ and 2B4^+^) on CD8+ TILs from B16F10-OVA tumors. nontreated *n* = 7, vehicle control *n* = 8; aPD1 *n* = 4; ETP-69 *n* = 9; ETP-69 + aPD-1 *n* = 10. **G** Pie chart of co-expression (PD-1^+^, TIM-3^+^, LAG-3^+^ and 2B44^+^) on CD8+ TILs from B16F10-OVA tumors. nontreated *n* = 7, vehicle control *n* = 8; aPD1 *n* = 4; ETP-69 *n* = 9; ETP-69 + aPD-1 *n* = 10. **H** Representative histogram and frequency (%) of GZMb^+^ and IFNγ^+^ cells among CD8+ TILs. Cells were re-stimulated in vitro with PMA and ionomycin for 4 h. nontreated *n* = 8, vehicle control *n* = 7; aPD1 *n* = 4; ETP-69 *n* = 10; ETP-69 + aPD-1 *n* = 10. **I** Representative histogram and frequency (%) of Ki67^+^ among CD8+ TILs from B16F10-OVA tumors. nontreated *n* = 7, vehicle control *n* = 8; aPD1 *n* = 4; ETP-69 *n* = 9; ETP-69 + aPD-1 *n* = 10. **J** Frequency (%) of TBET^+^EOMES^+^ among CD8^+^ PD-1^+^ TILs from B16F10-OVA tumors. non treated *n* = 7, vehicle control *n* = 8; aPD1 *n* = 4; ETP-69 *n* = 9; ETP-69 + aPD-1 *n* = 10. A representative experiment out of two is shown. *p* values were calculated using two-tailed Mann–Whitney test. *p < 0.05; **p < 0.01; ***p < 0.001; ****p < 0.0001. In all graphs, mean ± s.e.m. are presented. See also Supplementary Fig. 6. Source data are provided as a Source Data file.

Since genetic deficiency for Suv39h1 increases the response to anti-PD-1 treatment, we next evaluated the effect of ETP-69, a pharmacological inhibitor of Suv39h1 activity. ETP-69, which is not strictly specific for Suv39h1 (it also inhibits less potently G9A, another mono- and di-methylase of H3K9) has been previously reported to function in mice^39,40^. Here, we evaluated its effect in combination therapy with PD-1-blockade. For this, C57BL/6 mice bearing established B16F10-OVA tumors were treated daily with ETP-69 combined or not with anti-PD-1 (Fig. 6A). ETP-69 and anti-PD-1 administered as monotherapies induced a clear delay in tumor growth, and the combination of both was more effective than either of the single agents (Fig. 6B, C). The increased efficiency of the combination treatment was ETP-69 dose-dependant (Supplementary Fig. 6A, B). We conclude that pharmacological inhibition of Suv39h1 augments the efficacy of anti-PD-1 blockade in this tumor model.

We observed that frequencies and absolute numbers of CD8+ TILs were increased in ETP-69 treated mice, as compared to control groups, especially after treatment with anti-PD-1 (Fig. 6D). Similar to the results obtained in Suv39h1-defecient mice, ETP-69 alone or in combination with anti-PD-1 induced an increase in the proportion of effector CD8+ TILs, paralleled by a reduction in the proportion of memory TIL populations (Fig. 6E). In blood and secondary lymphoid organs, ETP-69 treatment lead to a reduction of naïve CD8+ T cells and increased proportions of central and effector memory subsets (Supplementary Fig. 6C). Furthermore, CD8+ TILs from ETP-69 treated mice expressed multiple inhibitory checkpoint receptors (Fig. 6F, G), and higher proportions of GZMb and IFNγ upon restimulation (Fig. 6H), and of Ki67+ cells (Fig. 6I). Finally, as observed for Suv39h1-KO mice treated with anti-PD-1, a higher proportion of CD8+ PD-1+ TILs from ETP-69 + anti-PD-1 treated mice co-expressed EOMES and TBET (Fig. 6J). We conclude that, similar to the Suv39h1-genetic defect, treatment of WT tumor-bearing mice with the Suv39h1 inhibitor ETP-69 reduces tumor growth, especially in combination with anti-PD-1. The inhibitor also enhances tumor infiltration by effector CD8+ T cells that express high levels of multiple inhibitory checkpoints but with an increased effector cytotoxic phenotype. We also evaluated the effect of ETP-69 in the less immunogenic B16 F10 tumor (without OVA expression) (Supplementary Fig. 6E–G), and also observed that ETP-69 induces a delay on tumor growth. Furthermore, Kebin Liu’s team has recently reported a novel small molecule, F5446, which inhibits Suv39h1 enzymatic activity more specifically^41^. We then treated C57BL/6 mice bearing established B16F10-OVA tumors with daily doses of F5446 combined or not with anti-PD-1 (Supplementary Fig. 6H–J). We observed that monotherapies induced a small delay on tumor growth, and that the combination was more efficient, especially in increasing mice survival. These latter results using a more selective Suv39H1 inhibitor suggest that the efficacy of the combination relies at least partially on Suv39h1 inhibition. Overall, pharmacological inhibition phenotypically and functionally phenocopies the genetic defect of Suv39h1.

## Discussion

In the tumor microenvironment, conditioned by hypoxia, nutrient shortage and tumor factors, “chronic” activation of CD8+ T lymphocytes progressively leads to loss of their effector functions and unresponsiveness to further stimulation, a process often referred to as “final exhaustion”. Although the signaling pathways involved in the induction of exhaustion are incompletely understood, the transcriptional programs expressed in exhausted cells have been analyzed in some details. Induction and silencing of hundreds of genes are under the control of critical transcription factors such as IRF4, cMAF, NFAT, TOX, EOMES and T-bet and others^3,42^. Similar to any gene expression program, transcriptional regulation of exhaustion and re-invigoration upon immune checkpoint blockade can only be promoted under a precise control of chromatin accessibility at the corresponding loci. Here, we identify Suv39h1, the main H3K9 tri-methylase and a hallmark of facultative and constitutive heterochromatin^43,44^, as a key epigenetic positive regulator of functional exhaustion.

Based on our results, we propose a working model in which, Suv39h1 contributes to progression of TILs along the exhaustion pathway through heterochromatin-mediated silencing of IFN-I gene expression programs and pathways downstream of TCR-signaling, inhibiting the deployment of an effector program. Thus, as cells become exhausted, silencing of these gene expression programs establishes an epigenetic barrier that prevents effective reprograming by PD-1 blockade. In the absence of Suv39h1, this epigenetic barrier is incomplete and TCR-triggering in memory/effector cells leads to an alternative activation pathway that allows survival and differentiation into highly cytolytic effectors without reaching final exhaustion. This in turn allows more effective re-invigoration of exhausted TILs by anti-PD-1 Ab, and more efficient tumor rejection. Previous studies have established that the chromatin changes in exhausted T cells upon anti-PD-1 treatment do not correspond to full reprogramming to, e.g. a memory phenotype^6,14^ and our results are consistent with these findings. We extend this, however, by showing that at the population level, response to anti-PD-1 is asymmetrically associated with chromatin opening, rather than closing, and is strongly associated with AP-1 factor-binding motifs suggestive of increased TCR signaling, effect which is much stronger in the absence of Suv39h1 activity.

How then does Suv39h1 repress TCR activation, terminal differentiation and ISG expression programs? Our previous work showed that several stem cell/memory-related genes display reduced levels of H3K9me3 deposition in *Suv39h1*-KO T cells, suggesting that they are direct targets of Suv39h1^10^. Chromatin analyses in TILs, however, are complicated because of reduced cell numbers and heterogeneity among individual mice. Interestingly, enhanced IFN-I signature genes are also overexpressed in hematopoietic stem cells^45^, suggesting a shared link between the mechanisms that control the two gene expression programs. The link could be direct, such as through deposition of the same histone marks (including H3K9me3) at memory and ISGs loci. H3K9me3 could also control ISGs indirectly, for example through regulation of expression of transposable elements (TEs), which in turn drive IFN-signaling. Indeed, Suv39h1 was shown to control expression of several families of TEs in fibroblasts and RNA from TEs can be sensed in the cytosol and induce activation of STING and IFN-I^46^.

To investigate the mechanisms of Suv39h1 action, we sequenced single cell transcriptomes from over 21,000 infiltrating CD8+ T cells from B16F10-OVA tumors in WT, or Suv39h1-defective mice, treated or not-treated by anti-PD-1 Ab. Our single cell analysis captured a high heterogeneity of CD8+ TILs, encompassing lymphocytes undergoing different states of activation: cycling, memory, memory effectors, progenitor exhausted, recently-TCR activated, IFN-responding, and cytolytic effectors subpopulations; but as expected no naïve cells. The frequency of cycling cells detected by this technique was not increased in anti-PD-1-treated mice (WT or *Suv39h1*-KO, Fig. 4B), while, at least in the KO, anti-PD-1 induced increased proportions of Ki67-positive cells. This could be due to limitations of the available transcriptomic signatures of cycling, as in our single cell analysis, the cluster of cycling cells includes cells in different phases of the G2M and S phases, which seem not to change in a systematic way among the different conditions (Supplementary Fig. 4B, C). The changes observed in the relative proportion of cells belonging to the other clusters indicate that during the anti-tumor response, Suv39h1 represses IFN-I signaling, and part of the exhaustion program, restraining the cells in a more memory/progenitor exhausted state, and T cell reinvigoration induced by PD-1 blockade drives these cells into a terminally exhausted program. However, in the absence of this epigenetic barrier imposed by Suv39h1, TCR-mediated reactivation is restored, and leads to highly cytolytic effectors with anti-tumor potential. Consistent with this working model, signatures from scRNAseq CD8+ T cells from melanoma patients that respond to immune checkpoint blockers correlated strongly with the transcriptomic signature of PD-1-treated *Suv39h1*-KO CD8+ T cells. Reciprocally, the PD-1-treated *Suv39h1*-KO signature was enriched in responding patients, indicating that this signature could potentially be used as biomarker of response in melanoma patients. Furthermore, the high granularity of CD8+ T cell populations characterized in scRNAseq analysis of the mouse data, allowed us to identify which were the specific CD8+ T subpopulations responding to ICB in patients, using as input published melanoma ICB response signatures obtained from single cell or bulk RNA sequencing. Of note, this analysis identified that, as in PD-1-treated *Suv39h1*-KO mice, response to immune checkpoint blockade was consistently associated to the emergence of cytolytic effector and TCR activation—a feature not previously highlighted in patients—and also IFN type I signatures, as previously described^36^.

Our results identify a critical epigenetic regulator of exhaustion. They also establish a clear link between IFN-I, early activation, cytolytic effectors and re-programing of progenitor exhausted cells. This link may be particularly relevant to the multiple immunotherapy approaches based on inducing IFN-I responses in the tumor environment, including cytolytic viruses, STING agonists, and DNA-demethylating agents. Since Suv39h1 genetic deficiency or pharmacological inhibition both re-activate anti-tumor immune responses, and promote re-programing by anti-PD-1, the role of Suv39h1 in this process must be at least partially non-redundant. By analogy to other non-redundant immunosuppressive proteins whose inhibition unleashes anti-tumor immune responses (often referred to as “immune checkpoints”), Suv39h1 can be considered as an “epigenetic immune checkpoint”. Should the role of Suv39h1 be conserved in human T cells, its blockade would open new perspectives for the epigenetic manipulation of anti-tumor T cell responses in the clinic.

## Methods

### Mice

C57BL/6J female and male mice (H-2^b^) (JAX#000664) and C57BL/6J female hybrids B6D2F1 [B6xDBA/]2 F1 (H-2^bxd^) (JAX#100006) mice were obtained from Charles River Laboratories (aged 8-10 weeks). Suv39h1^tm1Jnw^ C57BL/6J (*Suv39h1*-KO) mice were kindly provided by T. Jenuwein as previously described^47^, were backcrossed with C57Bl/6J mice for at least nine generations. C57BL/6J-Tg(*TcraTcrb*)1100Mjb/J mice (OT-I) (JAX#003831), and B6.SJL-*Ptprca Pepcb*/BoyJ (CD45.1) mice (JAX #002014) were purchased from Jackson lab. OT-I mice were crossed with CD45.1 mice to obtain OT-I/CD45.1 mice. Suv39h1^Flox/Flox^ (official name is B6-Suv39h1^tm1Ciphe^) were originally produced at the Centre d’Immunologie de Marseille (CIPHE, B. Malissen) and were subsequently bred in the animal facility of Institut Curie. The strategy targets the exon 2 from the transcript Suv39h1-001 ENSMUST00000115638. LoxP sites were introduced in the intron 1 at 70pb in 5′ of exon 2 and in the intron 2 at 98 pb in 3’ of exon 2. The loxP site in the intron found at the 3′ end of exon 2 was abutted to a Frt-neoR-Frt cassette. After deletion of the Frt-neoR-Frt cassette by flipage, a residual Frt site remain just after the loxP 3’. CD4-Cre transgenic mice were obtained from Jacques Ghysdaël (Institut Curie). *Suv39h1*-Flox*CD4-Cre^+/−^ and control *Suv39h1*-Flox*CD4-Cre^−/−^ mice were used for the experiment. Male mice, of either *Suv39h1*-KO, OT-1/CD45.1 and Suv39h1^Flox/Flox^ mouse strains were used aged 8–12 weeks. All animal procedures were in accordance with the guidelines and regulations of the Institut Curie veterinary department. Animal care and use for this study were performed in accordance with the recommendations of the European Community (2010/63/UE) for the care and use of laboratory animals. Experimental procedures were specifically approved by the ethics committee from Institut Curie, officially registered as CEEA-IC #118 and the Ministère de l’enseignement supérieur, de la recherche et de l’innovation which validated the project with the reference (APAFIS#12325_20171124123634-v2) in compliance with the international guidelines. Mice used in the experiments were age- sex-matched and were euthanized by cervical dislocation. Mice breeding were in SPF animal facilities and experimental and control animals were co-housed with housing conditions using a 12 light/12 dark cycle, with a temperature between 20 and 24 °C with an average humidity rate between 40% and 70%. Human endpoints were used for tumor-bearing mice as maximal ethical size of tumors subcutaneously grafted of 2 cm^3^, more than 20% of weight loss, signs of altered mobility-eating ability and cachexia.

### GVHD and leukemia model

Recipient B6D2F1 female mice received a 10-Gy irradiation followed by retro-orbital infusion of 5 × 10^6^ bone marrow cells (C57BL/6J) + 1 × 10^6^ CD3^+^ T cells from WT littermate or *Suv39h1*-KO male mice + 2 × 10^4^ mastocytoma cells P815-GFP derived from DBA/2 (H-2^d^) mice (gift from Dr. B. Salomon). Total splenocytes and lymph nodes suspension cells were prepared for CD3^+^ T cells enrichment using pan T cells isolation kit (Miltenyi). Bone marrow suspension were prepared using leg bones. Control groups were constituted of irradiated mice receiving only bone marrow or bone marrow and P815-GFP cells. GVHD and Leukemia was evaluated two times per week. GVHD was evaluated as previously described^48,49^, and leukemia by flow cytometry (GFP^+^ H-2K^d +^cells) and tumor bearing eyes.

### In vivo tumor progression, immunotherapy, and small molecule inhibitors

Half millon of melanoma cells B16F10, B16F10-expressing OVA (B16F10-OVA), MCA-101 fibrosarcoma cells, or one millon of EL4 lymphoma cells expressing OVA (EL4-OVA) were injected subcutaneously (s.c.) into the right flank of mice. Mice were randomly assigned a treatment group and tumor volume determined by caliper measurements. Anti-PD-1 Ab (clone RMP1-14, BioXcell), was administered intraperitoneally at 150 μg/20 g mice weight per dose on days 6–7, 11, 15 and 18–19. ETP-69 inhibitor was prepared as described previously^38^. Compound was dissolved in a mixture of Methyl cellulose 1% (Merck) and Poloxamer 188 0.1% (BASF Pharma Solutions) in water and administrated orally (20 mg/kg) once a day during two weeks to C57BL/6J female mice. F5446 inhibitor was kindly provided by Dr. Kebin Liu (Augusta, GA, USA) and administrated as described in Lu et al.^40^. Compound was dissolved in 10% Cremophor (Sigma) in PBS and injected intraperitoneally (10 mg/kg) every 2 days for a total of 7 doses to C57BL/6J female mice.

### Flow cytometry

For flow cytometric analysis, blood cells were harvested on day 12 post tumor implantation while DLN, spleen and tumor were harvested at day 19–20 post tumor implantation. Red blood cells were lysed with hypotonic buffer, single-cell suspensions were prepared in PBS 0.5% BSA and 2 mM EDTA (FACS buffer). Inguinal DLN and spleen were collected in CO_2_ Independent medium (GIBCO). Single-cell suspensions were obtained by mechanical disruption over a 40 μm cell strainer. Tumors were digested in 2 ml of CO_2_ Independent medium containing 0.1 mg/ml DNase I and 0.1 mg/ml Liberase TL (Roche) at 37 °C for 30 min in agitation. Samples were transferred to C tubes (Miltenyi Biotec) for mechanical dissociation with GentleMACS and cell suspension was then filtered with a 100 μm cell strainer. Mononuclear cells were recovered from Percoll gradient (GE Healthcare Life Science) from 40% to 75% interface, washed and resuspended in FACS buffer. Live/dead cell discrimination was performed using Live/Dead Fixable Aqua Dead Cell Stain Kit (Life Technologies) or DAPI (Life Technologies) and Fc receptors were blocked with the CD16/CD32 (clone 2.4.G2) mAb (BD) as per the manufacter’s instructions. Staining was performed with Abs listed in Supplementary Table 1. For intracellular staining of cytokines, cells were re-stimulated with phorbol 12-myristate 13-acetate (PMA, 20 ng/ml) and 1 μg/ml of ionomycin (Sigma-Aldrich) for 4 h at 37 °C in the presence of GolgiStop and GolgiPlug (BD Biosciences). Cell surface labelling was done according to the experiment at 4 °C and intracellular staining was done using a fixation/permeabilization kit (eBioscience/Thermo Fischer) according to the manufacturer’s instructions. Annexin V APC Apoptosis Detection Kit with 7-AAD (Ozyme) staining was performed after cell surface staining per the manufacturer’s instructions. All data acquisition was done using an LSRFortessa instrument with FACSDiva software v8.0.1 (BD) and analyzed using FlowJo software (TreeStar). Representative flow cytometry gating strategy for CD8+ T cells are shown in Supplementary Fig. 7A.

### Adoptive transfer

Total splenocytes and lymph nodes suspension cells of WT littermate and *Suv39h1*-KO CD45.1 OT-1 male mice were cultured for 6 days in vitro with anti-CD3 (10 μg/mL), anti-CD28 (1 μg/mL) mAbs (BD) and OVA peptide (SIINFEKL, 1 mg/ml) supplemented with rhIL-2 (100 U/mL Proleukin, Novartis) in RPMI medium (GIBCO), with 2-mercaptoethanol, Pen-Strep, l-Glutamine (LifeTechnologies), and 10% FCS (Biosera). Three million of treated cells/mouse were injected i.v. into previously EL4-OVA tumor inoculated C57BL/6J male mice and measured for tumor volume growth.

### Analysis of OVA-specific CD8+ T cells

Blood cells were harvested on day 12 post tumor implantation. 300,000 cells were plated per well in plates (MAIPS4510, Millipore) coated with anti-murine IFNγ Ab (Diaclone) and cultured overnight at 37 °C in RPMI medium (GIBCO), with 2-mercaptoethanol, Pen-Strep, l-Glutamine (LifeTechnologies), and 10% FCS (Biosera) with OVA peptide (SIINFEKL, 1 mg/ml) as antigen for restimulation. The IFNγ-producing cell were detected with biotinylated anti-IFNγ (Diaclone) followed by streptavidin-alkaline phosphatase (Mabtech) and revealed using the appropriate substrate (Biorad). Dots were counted with an ELISPOT Reader System ELR02 (AID, Germany). Tumor infiltrating cells, 20 days after tumor implantation, were stained with a mix of PE-conjugated H-2Kb/SIINFEKL and APC-conjugated H-2Kb/SIINFEKL tetramer, the biotiniylated monomer H-2Kb/SIINFEKL was produced by the recombinant protein facility P2R (Inserm-U1232, SFR Santé, Nantes, France) and analyzed using FlowJo software. The tetramer+ cells were gated on CD45+ TCRb+ CD8+ cells.

### Sample preparation for single-cell RNA-seq and ATAC-seq

*Suv39h1*-KO and littermate WT male mice were injected with B16-OVA tumor cells as previously described. On day 20, tumors were processed with Liberase and DNase I, stained with DAPI, CD45.2, TCRb, CD8, CD19, NK1.1, F4/80 and sorted on an FACSAria III (BD) by gating on live, CD45.2+, TCRb+, (CD19, NK1.1, F4/80-negative cells) and CD8+ on PBS 0.04% BSA. Representative flow cytometry gating strategy for sorting tumor-infiltrating of CD8+ T cells are shown in Supplementary Fig. 7B.

### Single-cell RNA-seq

Cells were loaded on a 10× Chromium instrument (10× Genomics) and libraries were prepared using a Single Cell 3′ Reagent Kit (V2 chemistry) (10× Genomics) according to manufacturer’s protocol. Briefly, the initial step consisted in performing an emulsion where individual cells were isolated into droplets together with gel beads coated with unique primers bearing 10× cell barcodes, unique molecular identifiers (UMI), and poly (dT) sequences. Reverse transcription reactions were engaged to generate barcoded full-length cDNA followed by the disruption of emulsions using the recovery agent and cDNA clean up with DynaBeads MyOne Silane Beads (Thermo Fisher Scientific). Amplification of bulk cDNA was achieved using GeneAmp PCR System 9700 with 96-Well Gold Sample Block Module (Applied Biosystems) (98 °C for 3 min; cycled 14×: 98 °C for 15 s, 67 °C for 20 s, and 72 °C for 1 min; held at 4 °C). A final clean-up step of amplified cDNA product was performed using the SPRI select Reagent Kit (Beckman Coulter). Indexed libraries were constructed following these steps: (1) fragmentation, end repair and A-tailing; (2) size selection with SPRI select beads; (3) adaptor ligation; (4) post-ligation cleanup with SPRI select beads; (5) sample index PCR and final cleanup with SPRI select beads. Library quantification and quality assessment were achieved by Qubit fluorometric assay (Invitrogen) using dsDNA HS (High Sensitivity) Assay Kit and Bioanalyzer Agilent 2100 System using a High Sensitivity DNA chip. Indexed libraries were tested for quality, equimolarly pooled and sequenced on an Illumina HiSeq2500 using paired-end 26 × 98bp as sequencing mode. By using a full Rapid flow cell, coverage was around 100M reads per sample corresponding to 100,000 reads per cell.

### Single-cell RNA-seq analysis

Single-cell expression was analyzed using the Cell Ranger Single Cell Software Suite (v2.0.1) to perform quality control, sample de-multiplexing, barcode processing, and single-cell 3′ gene counting. Sequencing reads were aligned to the mm10 v1.20 transcriptome using the Cell Ranger suite with default parameters. Downstream analyses were performed using Seurat (v3.2.1)^50^. Starting from the unfiltered matrices, empty droplets were discarded using the EmptyDrops package^51^ using a FDR threshold of 0.01. The gene-cell-barcode matrix of individual samples was log-transformed and filtered based on the number of genes detected per cell (any cell with <500 genes or more than 5000 genes per cell was filtered out). Based on inspection of the UMI distribution over mitochondrial content, any cell with more than 9% of mitochondrial UMI counts. Contaminating cells were removed using the expression of Tyrobp, H2-Eb1, Apoe and Hbb-b. After filtering, a total of 21,646 single cells were analyzed. To allow comparison between genotypes, treatments and replicates, integration was performed using Seurat v3 anchors procedure, selecting for the 2000 most variable genes using the ‘vst’ method and computing anchors using the first 30 principal components. Clusters were then identified using the “Find_Clusters” function in Seurat with a resolution parameter of 0.5. Clustree analysis was performed using Clustree R package (Version 0.2.2)^52^. 16 clusters were initially defined, and are visualized using UMAP in Supplementary Fig. 4A. To simplify the analysis, some clusters which shared defined signatures and which did not evidently change among the different conditions were grouped as follow: original clusters 1, 3, 6, 7 and 11, corresponded to cycling cells in the G1/S and G2/M phase (Supplementary Fig. 4B, C), were merged into one cluster (cluster 8 “cycling cells”, Fig. 3B); and original clusters 0, 8, 12, 13, and 14 (Supplementary in Fig. 4A) which shared a “late exhaustion” signature, were merged together (as cluster 7 “late exhausted”, Fig. 3B). Unique cluster-specific genes were identified by running the Seurat “Find_All_Markers” function using the wilcoxon test. Signature scores were computed using the Seurat function “AddModuleScore” using default parameters.

### ATAC-seq

Chromatin profiling was performed by ATAC-seq as described in Buenrostro et al. ^37^. In brief, 50,000 cells per sample were washed in cold PBS and lysed. Transposition reaction was performed at 37 °C for 30 min and DNA was purified with MinElute PCR purification kit (Qiagen). Transposed DNA was amplified for 5 cycles and additional PCR cycles were evaluated by real-time PCR. Libraries were sequenced on Novaseq 6000 using 1xSp-Flow Cell (Illumina). A total of 130–210 × 10^6^ paired reads were generated per sample.

### ATAC-seq data analysis

(1) *Alignment, peak calling, and visualization*: ATAC-Seq sequencing reads were aligned to the mouse reference genome mm10 using BWAMEM (bwa-0.7.15, opt -k 19 -T 30 -M). Only properly paired uniquely mapped reads were kept for further analysis (samtools -f 0 × 2 -q 30). Peaks were called using MACS2 v2.1.2 with default parameters and a 5% FDR threshold. ATAC-seq signal tracks were generated using bamCoverage (deeptools-v2.5.3, –binSize 10 –normalizeUsing BPM), and visualized in Integrative Genomics Viewer (IGV) software. (2) *Comparison between conditions*: Two strategies of peak calling were performed: (1) to detect individual peaks in each separate condition, peaks were called on merged replicates versus genome background; (2) to detect modulated peaks between conditions, peaks were called on merged replicates using 1 condition as treatment and another as control; further analysis on modulated peaks were filtered either by (1) only real peaks identified above by the peak caller, (2) fold-enrichment ≥ =2, or (3) uniquely identified in treatment condition during individual peak calling. (3) *Signature enrichment analysis*: ATAC peaks were annotated using HOMER::annotatePeaks to nearest TSS gene. Signature enrichment analysis was applied on selected signatures using hypergeometric test alternative greater and BH adjusted *p*-value < 0.01. Transcription factor motif enrichment was used to identify transcriptional programs associated with the epigenetic changes.

### Statistical analysis

Data are given as mean ± s.e.m. Prism software (Graphpad Software, Inc.) was used to perform statistical analysis. Two-tailed Mann–Whitney test was used to compare groups statistically. Long-rank (Mantel–Cox) test was performed for statistical analysis of survival study. A *p* value lower than 0.05 (*p < 0.05) was considered as threshold for statistical significance among control groups and experimental groups.

### Reporting summary

Further information on research design is available in the Nature Research Reporting Summary linked to this article.

## Supplementary information

Supplementary Information

Reporting Summary

## Data Availability

The scRNAseq and ATAC-seq data generated in this study have been deposited in the Gene Expression Omnibus repository under accession code GSE198423. Source data are provided with this paper.

## Source data

Source Data
