# Author Correction: CD8+T cell responsiveness to anti-PD-1 is epigenetically regulated by Suv39h1 in melanomas

**DOI:** 10.1038/s41467-023-38931-6

**Published:** 2023-05-30

**Authors:** Leticia Laura Niborski, Paul Gueguen, Mengliang Ye, Allan Thiolat, Rodrigo Nalio Ramos, Pamela Caudana, Jordan Denizeau, Ludovic Colombeau, Raphaël Rodriguez, Christel Goudot, Jean-Michel Luccarini, Anne Soudé, Bruno Bournique, Pierre Broqua, Luigia Pace, Sylvain Baulande, Christine Sedlik, Jean-Pierre Quivy, Geneviève Almouzni, José L. Cohen, Elina Zueva, Joshua J. Waterfall, Sebastian Amigorena, Eliane Piaggio

**Affiliations:** 1 Institut Curie, PSL Research University, F-75005 Paris, France; 2 INSERM U932, F-75005 Paris, France; 3 Translational Research Department, Institut Curie, F-75005 Paris, France; 4 Université Paris-Est, UMR S955, Université Paris-Est Créteil Val de Marne, Créteil, France; 5 INSERM, U955, Equipe 21, Créteil, France; 6 Institut Curie, PSL Research University, CNRS UMR3666, INSERM U1143, Chemical Biology of Cancer, Equipe Labellisée Ligue contre le Cancer, Paris, France; 7 Inventiva, 50 rue de Dijon, 21121 Daix, France; 8 Institut Curie, Genomics of Excellence (ICGex) Platform, Institut Curie Research Center, Paris, France; 9 Institut Curie, PSL Research University, CNRS, UMR3664, Equipe Labellisée Ligue contre le Cancer, Paris, France; 10 Sorbonne Universités, UPMC University Paris 06, CNRS, UMR3664, F-7005 Paris, France; 11 INSERM U830, F-75005 Paris, France

**Keywords:** Immunotherapy, Cancer immunotherapy, Histone post-translational modifications, Lymphocyte activation

Correction to: *Nature Communications* 10.1038/s41467-022-31504-z, published online 29 June 2022

The original version of this Article contained an error in Figure 4. The lower panel image of Figure 4A was missing in the original version.

This error has been corrected in both the PDF and HTML versions of the Article.

