# Supplementary material for: CD8+T cell responsiveness to anti-PD-1 is epigenetically regulated by Suv39h1 in melanomas
Source: Nat Commun. 2022 Jun 29;13:3739. doi: 10.1038/s41467-022-31504-z (PMC9243005; doi:10.1038/s41467-022-31504-z)
Supplement: Supplementary file 1 — Supplementary Information [file 41467_2022_31504_MOESM1_ESM.pdf]

Supplementary Information File

CD8+ T cell responsiveness to anti-PD-1 is epigenetically regulated by Suv39h1 in melanomas

Leticia Laura Niborski[1,2,3], Paul Gueguen[1,2], Mengliang Ye[1,2], Allan Thiolat[4,5], Rodrigo Nalio Ramos[1,2,3], Pamela Caudana[1,2,3], Jordan Denizeau[1,2,3], Ludovic Colombeau[6], Raphaël Rodriguez[6], Christel Goudot[1,2], Jean-Michel Luccarini[7], Anne Soudé[7], Bruno Bournique[7], Pierre Broqua[7], Luigia Pace[1,2,8], Sylvain Baulande[9], Christine Sedlik[1,2,3], Jean-Pierre Quivy[1,10,11], Geneviève Almouzni[1,10,11], José L. Cohen[4,5], Elina Zueva[1,2], Joshua J. Waterfall[1,3,12], Sebastian Amigorena[1,2‡*] and Eliane Piaggio[1,2,3‡*]

1 Institut Curie, PSL Research University, F-75005 Paris, France.
2 INSERM U932, F-75005, Paris, France.
3 Institut Curie, Translational Research Department, F-75005 Paris, France.
4 Université Paris-Est, UMR S955, Université Paris-Est Créteil Val de Marne, Créteil, France.
5 INSERM, U955, Equipe 21, Créteil, France.
6 Institut Curie, PSL Research University, CNRS UMR3666, INSERM U1143, Chemical Biology of Cancer, Equipe Labellisée Ligue contre le Cancer, Paris, France
7 Inventiva, 50 rue de Dijon, 21121 Daix, France.
8 Armenise-Harvard Immune Regulation unit, IIGM, Candiolo (TO), 10060, Italy ; Candiolo Cancer Institute, FPO-IRCCS, Candiolo (TO), 10060, Italy.
9 Institut Curie, Genomics of Excellence (ICGex) Platform, Institut Curie Research Center, Paris, France
10 Institut Curie, PSL Research University, CNRS, UMR3664, Equipe Labellisée Ligue contre le Cancer, Paris, France.
11 Sorbonne Universités, UPMC University Paris 06, CNRS, UMR3664, F-7005 Paris, France.
12 INSERM U830, F-75005 Paris, France.

‡Equally contributed authors
***Corresponding authors:**
sebastian.amigorena@curie.fr
eliane.piaggio@curie.fr

Content

Supplementary Figure 1. *Suv39h1*-KO CD8+ T cells show higher anti-tumoral effects than WT CD8+ T cells. Related to Figure 1.

Supplementary Figure 2. *Suv39h1* is critical for peripheral CD8+ T cell differentiation to a memory like phenotype. Related to Figure 2.

Supplementary Figure 3. *Suv39h1*-KO mice treated with anti-PD-1 show CD8+ TILS with altered effector and exhaustion phenotypes. Related to Figure 2.

Supplementary Figure 4. Characterization of CD8+ TILs clusters by scRNAseq. Related to Figure 3 and Figure 4.

Supplementary Figure 5. Transcriptomic correlation between human and *Suv39h1*-KO mice responses to immune checkpoint blockade. Related to Figure 4.

Supplementary Figure 6. Pharmacological inhibition of Suv39h1 increases tumor rejection

Supplementary Figure 7. Gating strategies for flow cytometric analysis and cell sorting.

Supplementary Table 1. List of antibodies used on this study.

Supplementary Fig.1

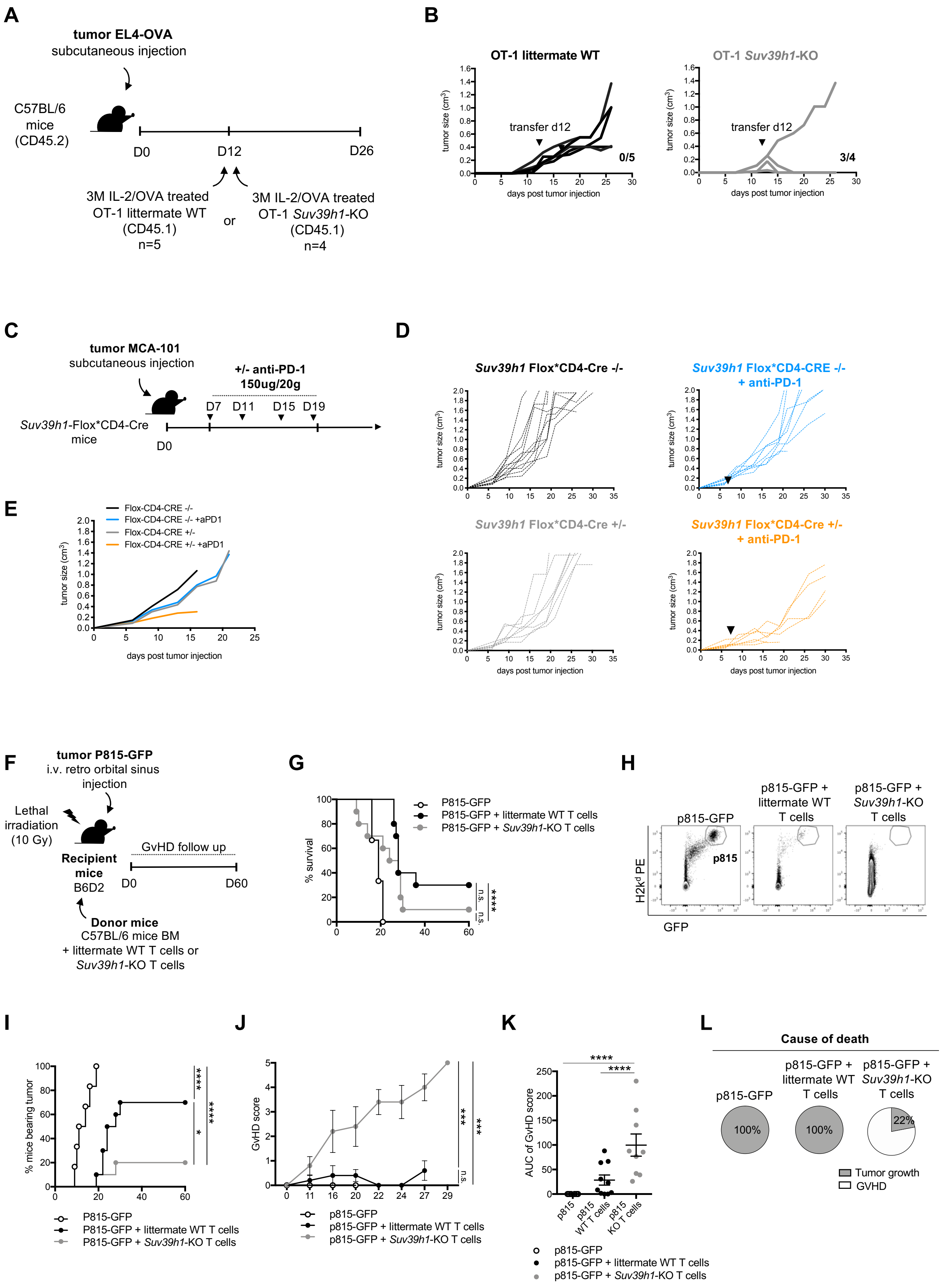

Supplementary Figure 1. *Suv39h1*-KO CD8+ T cells show higher anti-tumoral effects than WT CD8+ T cells. Related to Figure 1.

(A) EL4-OVA tumor model. Experimental design. Littermate WT or Suv38h1-KO OT-I cells (CD45.1+) were activated with IL-2 and OVA-I peptide for 6 days and adoptively transferred (i.v.) to congenic CD45.2+ recipient mice bearing EL4-OVA tumor.

(B) Tumor growth kinetics. Numbers refer to rejected tumors out of total mice analyzed.

(C) Scheme of the treatment in Suv39h1-Flox*CD4-Cre +/- mice and Suv39h1-Flox*CD4-Cre -/- control littermates receiving MCA-101 fibrosarcoma cells followed by PBS or anti-PD-1 Ab.

(D) Individual mouse tumor growth kinetics curves. Black arrows indicate time point of initial anti-PD-1 Ab injection.

(E) Tumor growth kinetics curves represented as mean. Suv39h1-Flox*CD4-Cre -/- mice PBS (n=11), Suv39h1-Flox*CD4-Cre -/- mice (+) anti-PD1 (n=8), Suv39h1-Flox*CD4-Cre +/- PBS (n=7) and Suv39h1-Flox*CD4-Cre +/- (+) anti-PD-1 (n=6). Results were obtained in one experiment.

(F) P815-GFP tumor model. P815-GFP cells were injected into irradiated B6D2F1 recipient mice grafted with bone marrow cells (n=6), supplemented with littermate WT (n=10) or Suv39h1-KO (n=10) CD3+ T cells. The experiment was performed twice and the resulting survival (G), tumoral incidence (H-I) and clinical score (J-K) data were pooled. The pie chart (L) represent the proportion of mice dead of leukemia (grey) or GvHD (white). Kaplan-Meier survival curves were compared using log-rank test. For analysis of GvHD clinical grading curves, AUC was calculated for each mouse, and then Student t test or 1-way ANOVA with post hoc analysis was performed depending on number of comparatives. ns, nonsignificant. * p<0.05; **** p<0.0001. **** p<0.0001. Source data are provided as a Source Data file.

Supplementary Fig.2 (related Fig.1)

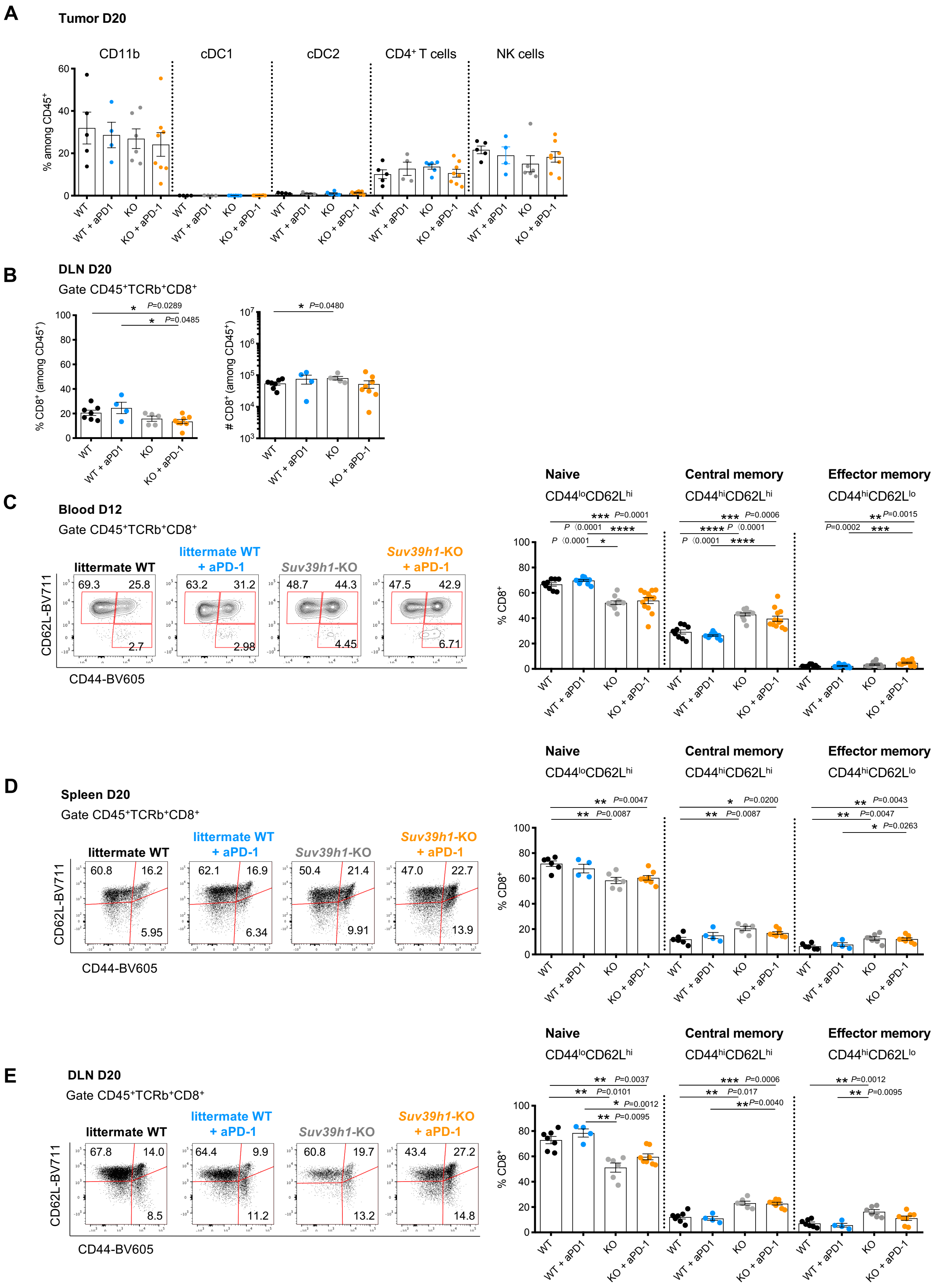

Supplementary Figure 2. ***Suv39h1*** **is critical for peripheral CD8+ T cell differentiation to a memory like phenotype. Related to Figure 2.**

(A) Frequency (%) of CD11b+, cDC1 ($CD45^+CD64^-F4/80^+Lin^-$(CD19, NK1.1, TCRb) $MHCII^+CD11C^+CD26^+XCR1^+CD127a^-$), cDC2 ($CD45^+CD64^-F4/80^+Lin^-$(CD19, NK1.1, TCRb) $MHCII^+CD11C^+CD26^+XCR1^-CD127a^+$) and NK cells ($CD45^+TCRb^-NK1.1^+$), in B16F10-OVA tumors at day 20 after tumor inoculation. CD11b: WT *n*=5; WT + aPD-1 *n*=4; KO *n*=6; KO + aPD-1 *n*=8; cDC1: WT *n*=4; WT + aPD-1 *n*=4; KO *n*=6; KO + aPD-1 *n*=8; cDC2: WT *n*=4; WT + aPD-1 *n*=4; KO *n*=6; KO + aPD-1 *n*=8; $CD4^+$ T cells: WT *n*=5; WT + aPD-1 *n*=4; KO *n*=6; KO + aPD-1 *n*=8; NK cells: WT *n*=5; WT + aPD-1 *n*=4; KO *n*=6; KO + aPD-1 *n*=8.

(B) Frequency (%) and quantification (number) of $CD8^+$ in DLN at day 20 after tumor inoculation ($CD45^+TCRb^+CD4^-$). WT *n*=7; WT + aPD-1 *n*=4; KO *n*=5; KO + aPD-1 *n*=8.

(C) Representative contour plots and frequency (%) of CD8+ naive, central memory and effector T cells in blood from tumor bearing mice at day 12 after tumor inoculation. WT *n*=9; WT + aPD-1 *n*=12; KO *n*=11; KO + aPD-1 *n*=13.

(D) Representative dot plots and frequency (%) of CD8+ naive, central memory and effector T cells in spleen from tumor bearing mice at day 20 after tumor inoculation. WT *n*=6; WT + aPD-1 *n*=4; KO *n*=6; KO + aPD-1 *n*=8.

(E) Representative dot plots and frequency (%) of CD8+ naive, central memory and effector T cells in DLN from tumor bearing mice at day 20 after tumor inoculation. WT *n*=7; WT + aPD-1 *n*=4; KO *n*=6; KO + aPD-1 *n*=8.

A representative experiment out of two is shown. In all graphs, mean±s.e.m. are presented. p values were calculated using two-tailed Mann-Whitney test. *p<0.05; **p<0.01; ***p<0.001; ****p<0.0001. Source data are provided as a Source Data file.

Supplementary Fig.3 (related Fig.2)

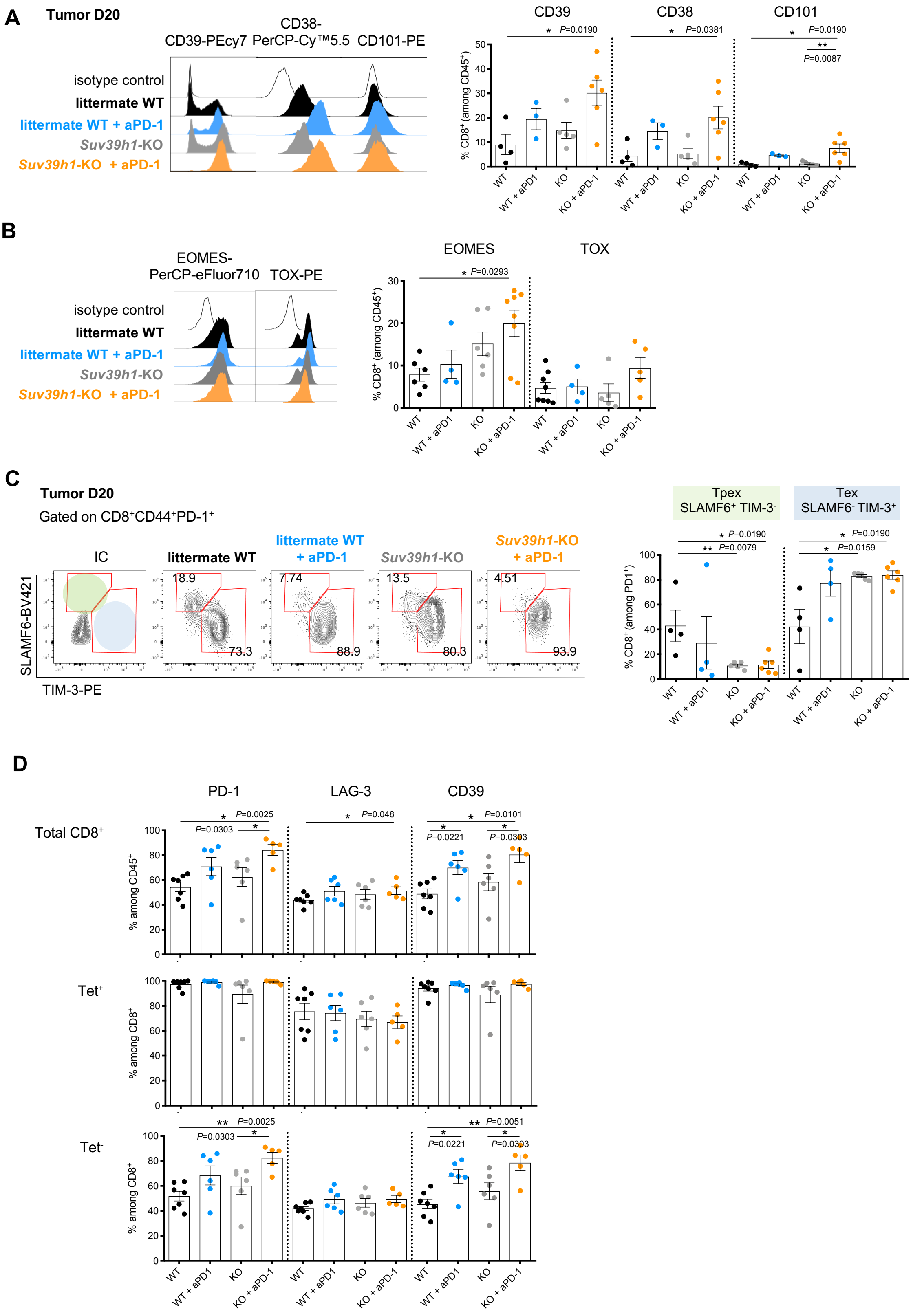

Supplementary Figure 3. *Suv39h1*-KO mice treated with anti-PD-1 show CD8+ TILS with altered effector and exhaustion phenotypes. Related to Figure 2.

(A) Representative histogram and frequency (%) of surface receptors ($CD39^+$, $CD38^+$ and $CD101^+$) on $CD8^+$ TILs from B16F10-OVA tumors. WT *n*=4; WT + aPD-1 *n*=3; KO *n*=5; KO + aPD-1 *n*=6.

(B) Representative histogram and frequency (%) of transcription factors EOMES and TOX on $CD8^+$ TILs from B16F10-OVA tumors. EOMES: WT *n*=6; WT + aPD-1 *n*=4; KO *n*=6; KO + aPD-1 *n*=6. TOX: WT *n*=8; WT + aPD-1 *n*=4; KO *n*=5; KO + aPD-1 *n*=5.

(C) Representative contour plots and frequency (%) of progenitor exhausted ($SLAMF6^+TIM\text{-}3^-$) and late exhausted ($SLAMF6^-TIM\text{-}3^+$) among $CD8^+$ PD-1^+ TILs from B16F10-OVA tumors. WT *n*=4; WT + aPD-1 *n*=4; KO *n*=5; KO + aPD-1 *n*=6.

(D) Frequency (%) of PD-1^+, LAG-3^+ and $CD39^+$ among total $CD8^+$ (upper panel) or among Tet^+ (middle panel) or Tet^- (lower panel) $CD8^+$ TILs. WT *n*=7; WT + aPD-1 *n*=6; KO *n*=6; KO + aPD-1 *n*=5.

A representative experiment out of two is shown. In all graphs, mean±s.e.m. are presented. p values were calculated using two-tailed Mann-Whitney test. *p<0.05; **p<0.01. Source data are provided as a Source Data file.

Supplementary Fig.4 (related Fig.3 and Fig.4)

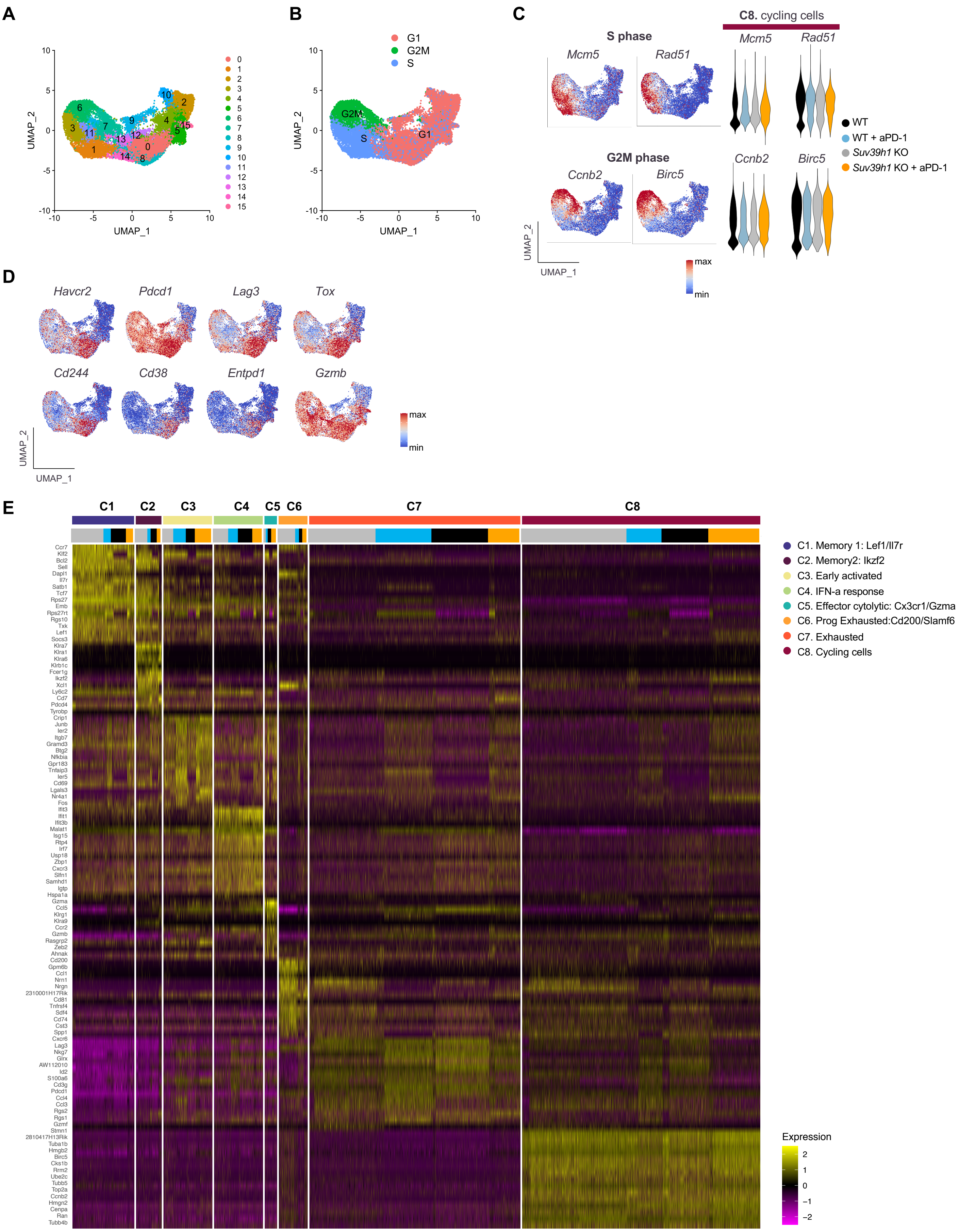

Supplementary Fig.4 continuation (related Fig.3 and Fig.4)

F

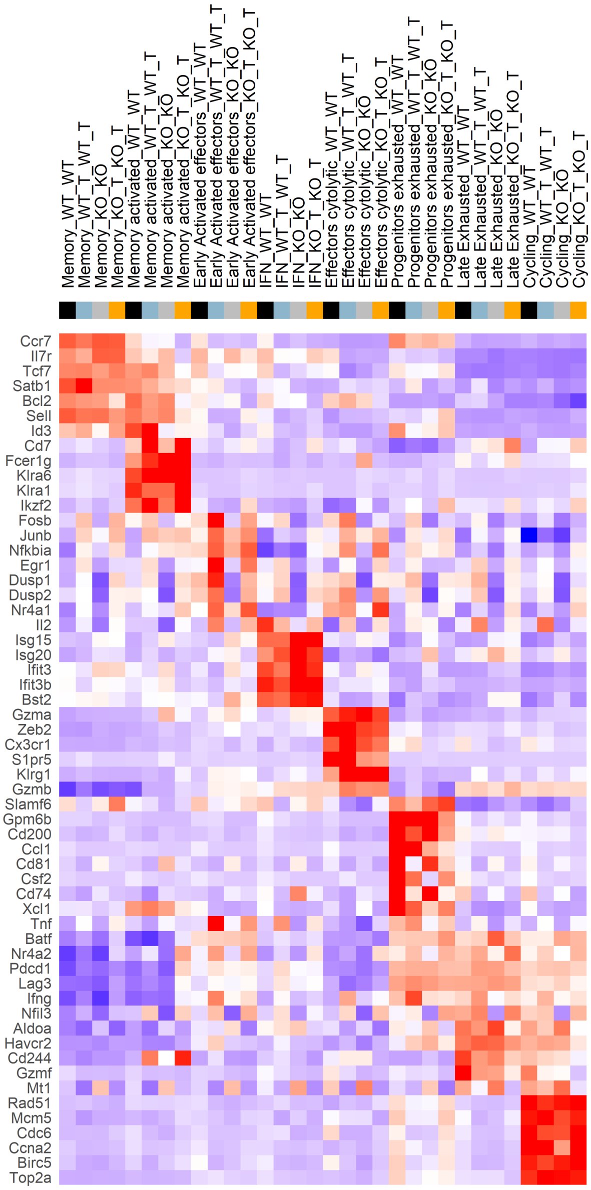

Supplementary Figure 4. Characterization of CD8+ TILs clusters by scRNAseq. Related to Figure 3 and Figure 4.

(A) UMAP plot displaying 16 clusters based on gene expression differences for 21646 passing quality control cells (resolution 0.7). Clusters with matched identities and equivalent CD8^{+} TILs proportions among littermate WT and *Suv39h1*-KO treated or not with anti-PD-1 were merged resulting in a final of 8 clusters (see Fig.3B).

(B) UMAP plot showing cell cycle phases.

(C) Feature and violin plots showing expression of cell cycle phase genes.

(D) Feature plots showing expression of indicated genes.

(E) Heatmap from single-cell analysis of 21646 cells from littermate WT and *Suv39h1*-KO treated or not with anti-PD-1 grouped into clusters. The top 15 genes differentially expressed for each cluster are shown in y axis. Heatmap colour scheme is based on Z-score distribution.

(F) Heatmap displaying genes that characterize the identified clusters across conditions.

Supplementary Fig.5 (related Fig.4)

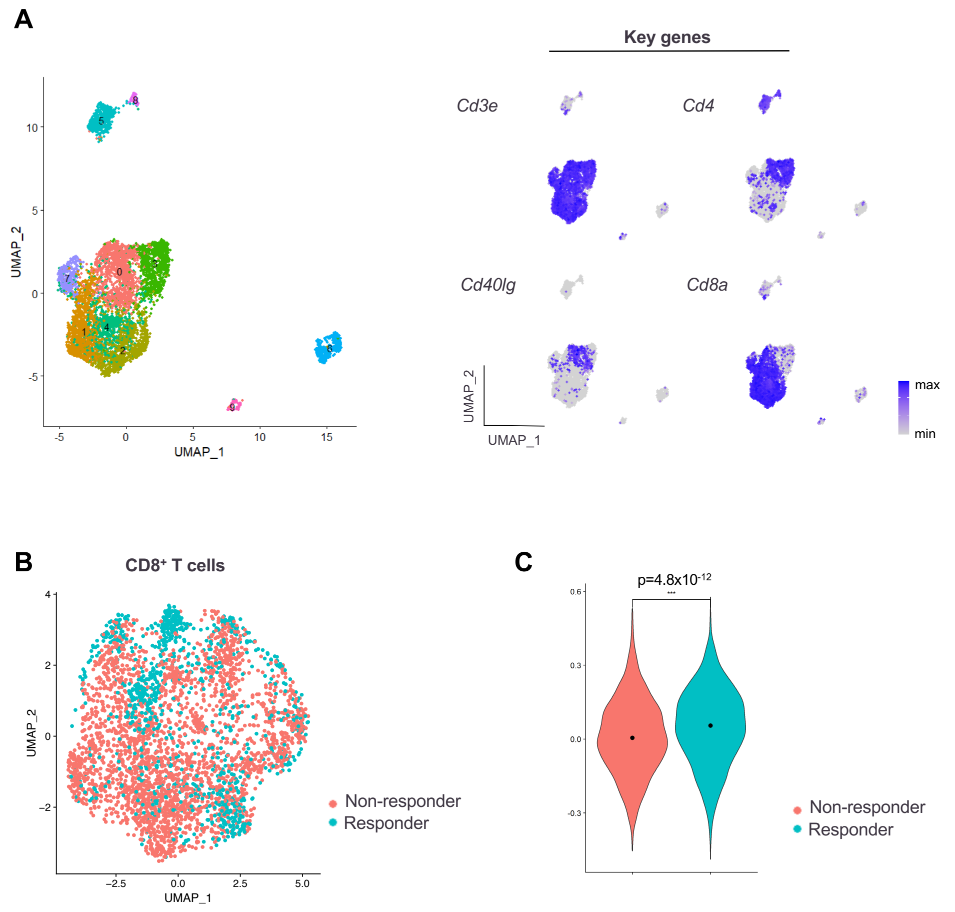

Supplementary Figure 5. Transcriptomic correlation between human and *Suv39h1*-KO mice responses to immune checkpoint blockade. Related to Figure 4.

(A) To more faithfully compare the human and mice signature of CD8$^+$ TILS we first reprocessed the human CD45$^+$ single cell data extracted from Sade-Feldman et al. 2018, to generate a new dataset containing only CD8$^+$ T cells. Left: UMAP plot displaying scRNAseq of all CD45$^+$ from melanoma patient responders and non responders to ICB treatment, before eliminating non CD8$^+$ T cells. Right: feature plots showing expression of indicated genes used to conserve CD8+ T cells.

(B) UMAP plot displaying CD8$^+$ T cells from melanoma patients and identifying CD8$^+$ T cells from patients responding or not to ICB treatment (Sade-Feldman et al., 2018).

(C) Violin plots showing the enrichment of signature of genes upregulated in *Suv39h1*-KO + aPD-1 signature projected in CD8$^+$ T cells from melanoma patient responders and non responders to ICB treatment (Sade-Feldman et al., 2018). p value was calculated using two-sided Wilcoxon rank-sum test.

Supplementary Fig.6 (related to Fig.6)

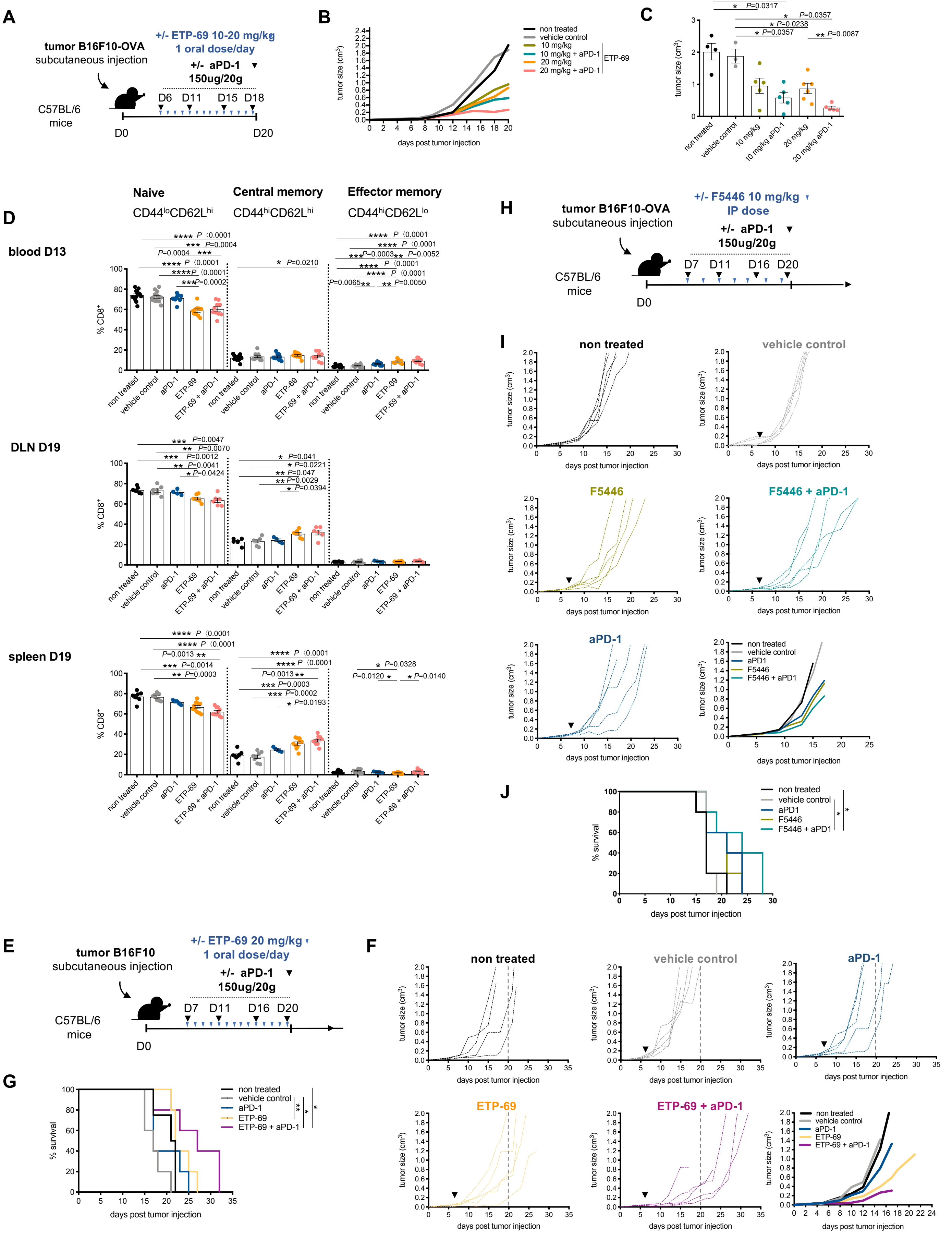

Supplementary Figure 6. Pharmacological inhibition of Suv39h1 increases tumor rejection. Related to Figure 6.

(A) Graphical representation of model system of experimental groups, including C57BL/6 mice receiving B16F10-OVA melanoma cells followed by ETP-69 oral treatment (10 mg/kg or 20 mg/kg), PBS or anti-PD-1 Ab injection.

(B) Tumor growth kinetics represented as mean of one independent experiment.

(C) Tumor volumes in cm3 (day 20). non treated *n*=4, vehicle control *n*=3, 10 mg/kg *n*=5; 10 mg/kg + aPD-1 n=5, 20 mg/kg *n*=6, 20 mg/kg + aPD-1 *n*=5. A representative experiment out of two is shown. In all graphs, mean±s.e.m. are presented. p values were calculated using two-tailed Mann-Whitney test. *p<0.05; **p<0.01.

(D) Frequency (%) of CD8+ naive, central memory and effector T cells in blood, DLN and spleen from tumor bearing mice at day 12 or day 19 after tumor inoculation. Blood: non treated *n*=14, vehicle control *n*=13, aPD-1 *n*=10, ETP-69 *n*=10, ETP-69 + aPD-1 *n*=5; DLN: non treated *n*=7, vehicle control *n*=13, aPD-1 *n*=4, ETP-69 *n*=7 , ETP-69 + aPD-1 *n*=6; spleen: non treated *n*=8, vehicle control *n*=8, aPD-1 *n*=5, ETP-69 *n*=10, ETP-69 + aPD-1 *n*=10. A representative experiment out of two is shown. In all graphs, mean±s.e.m. are presented. p values were calculated using two-tailed Mann-Whitney test. *p<0.05; **p<0.01; ***p<0.001; ****p<0.0001.

(E) Scheme of the treatment in C57BL/6 mice receiving B16F10 melanoma cells followed by ETP-69 oral treatment, PBS or anti-PD-1 Ab injection.

(F) Individual mouse curves of tumor growth kinetics. Black arrows indicate time of initial ETP-69 or vehicle control dose administration and anti-PD-1 Ab injection. Lower right panel shows tumor growth kinetics represented as means. non treated *n*=4, vehicle control *n*=5; aPD1 *n*=5; ETP-69 *n*=5; ETP-69 + aPD-1 *n*=5.

(G) Survival of B16F10 bearing mice subjected to the indicated treatments. Log-rank (Mantel-Cox) test was used for statistical analysis comparing the indicated groups with the non treated or vehicle control injected group. non treated vs. ETP-69 + aPD-1: *P=0.0473; vehicle control vs. ETP-69 + aPD-1: *P=0.0142; vehicle control vs. ETP-69: **P=0.0042. *p < 0.05; ** p < 0.01. Shown is one representative experiment out of 2 with similar results.

(H) Graphical representation of model system of experimental groups, including C57BL/6 mice receiving B16F10-OVA melanoma cells followed by PBS, vehicle control, F5446 IP treatment, and/or anti-PD-1 Ab injection. *n*=5 mice per group.

(I) Individual mouse curves of tumor growth kinetics. Black arrows indicate time of initial F5446 or vehicle control dose administration and anti-PD-1 Ab injection. Lower right panel shows tumor growth kinetics represented as means. *n*=5 mice per group.

(J) Survival of B16F10-OVA bearing mice subjected to the indicated treatments. Log-rank (Mantel-Cox) test was used for statistical analysis comparing the indicated groups with the non treated or vehicle control injected group. *n*=5 mice per group. non treated vs. F5446 + aPD-1: *P=0.0392; vehicle control vs. F5446 + aPD-1: *P=0.0298. *p < 0.05. Source data are provided as a Source Data file.

Supplementary Fig.7

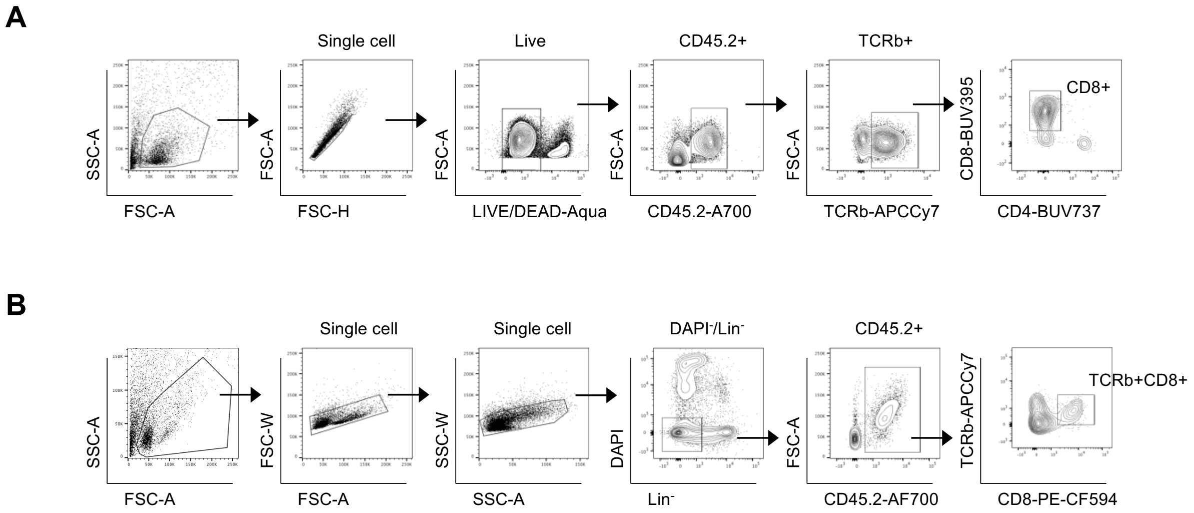

Supplementary Figure 7. Gating strategies for flow cytometric analysis and cell sorting.

(A) Representative flow cytometry gating strategy for tumor-infiltrating CD8+ T cells. Related to Fig.1, Fig.2 , Fig.6, Fig.S2, Fig.S3, and Fig.S6.

(B) Gating strategy for FACS-sorting of CD8+ T cells from tumors for scRNA-seq and ATAC-seq. Lineage (Lin-) includes CD19, NK1.1, F4/80 antibodies. Related to Fig.3, Fig.4, Fig.5, Fig. S4 and Fig.S5.

Supplementary Table 1. List of antibodies used on this study.

Antibody	Clone	Reference	Supplier	Color	Dilution
BCL-2	BCL/10C4	633508	Biolegend	PE	1:100
CD4	RM4-5	564933	BD	BUV737	1:200
CD4	GK1.5	563790	BD	BUV395	1:200
CD8a	53-6.7	563786	BD	BUV395	1:200
CD8a	53-6.7	564297	BD	BUV737	1:200
CD8a	53-6.7	562315	BD	PE-CF594	1:1600
CD11b	M1/70	101261	Biolegend	APC-Cy7	1:3200
CD11b	M1/70	101216	Biolegend	PE-Cy7	1:1000
CD11c	N418	MCD11C17	Invitrogen	PE-Texas Red	1:200
CD19	6D5	115520	Biolegend	PE-Cy7	1:1600
CD26	H194-112	137806	Biolegend	FITC	1:100
CD38	90/CD38	562770	BD	PerCP-Cy™5.5	1:200
CD39	24DMS1	25-0391-82	Invitrogen	PE-Cy7	1:100
CD44	IM7	103047	Biolegend	BV605	1:800
CD44	IM7	103049	Biolegend	BV650	1:400
CD45.2	104	109822	Biolegend	Alexa Fluor 700	1:1600
CD45.2	104	564880	BD	BUV737	1:100
CD62L	MEL-14	553152	BD	APC	1:1000
CD62L	MEL-14	740660	BD	BV711	1:1600
CD62L	MEL-14	533150	BD	FITC	1:1600
CD64	X54-5/7.1	139306	Biolegend	APC	1:100
CD101	307707	564473	BD	Alexa Fluor 647	1:100
CD172a (SIRP alpha)	P84	46-1721-82	Invitrogen	PerCP-eFluor 710	1:100
CD223 (LAG-3)	C9B7W	125221	Biolegend	BV421	1:100
CD244.2 (2B4)	eBio244F4	11-2441-82	eBioscience	FITC	1:100
CD279 (PD-1)	29F.1A12	135225	Biolegend	BV785	1:100
CD335 (NKp46)	29A1.4	25-3351-82	eBioscience	PE-Cy7	1:200
CD366 (Tim-3)	RTM3-23	119706	Biolegend	APC	1:100
CD366 (Tim-3)	RTM3-23	134004	Biolegend	PE	1:100
EOMES	Dan11mag	61-4875-80	eBioscience	PE-eFluor 610	1:100
EOMES	Dan11mag	46-4875-82	eBioscience	PerCP-eFluor 710	1:200
F4/80	BM8	123141	Biolegend	BV785	1:100
F4/80	BM8	25-4801-82	eBioscience	PE-Cy7	1:100
Granzyme B	GB11	515408	Biolegend	Pacific Blue	1:100
H-2K[b]	AF6-88.5	562832	BD	Alexa Fluor 647	1:50
ISG15	F-9	sc-166755	Sta Cruz Bio.	PE	1:200
IFNg	B27	564039	BD	BV711	1:100
Ki67	B56	556027	BD	PE	1:80
MHC Class II (I-A/I-E)	M5/114.15.2	48-5321-82	Invitrogen	eFluor 450	1:1000
NK1.1	PK136	108716	Biolegend	PE-Cy5	1:400
NK1.1	PK136	552878	BD	PE-Cy7	1:200
SLAMF6 (Ly-108)	13G3	740090	BD	BV421	1:100
Tbet	4B10	644824	Biolegend	PE-Cy7	1:400
TCF1	C63D9	6444S	Biolegend	Alexa Fluor 488	1:100
TCRb	H57-597	109220	Biolegend	APC-Cy7	1:400
TCRb	560729	566345	BD	PE-Cy7	1:200
TOX	TXRX10	12-6502-82	Invitrogen	PE	1:100
XCR1	ZET	148220	Biolegend	BV650	1:200
