# Supplementary material for: CD8+T cell responsiveness to anti-PD-1 is epigenetically regulated by Suv39h1 in melanomas
Source: Nat Commun. 2022 Jun 29;13:3739. doi: 10.1038/s41467-022-31504-z (PMC9243005; doi:10.1038/s41467-022-31504-z)
Supplement: Supplementary file 2 — Reporting Summary [file 41467_2022_31504_MOESM2_ESM.pdf]

nature research

Corresponding author(s): Eliane Piaggio

Last updated by author(s): Apr 24, 2022

Reporting Summary

Nature Research wishes to improve the reproducibility of the work that we publish. This form provides structure for consistency and transparency in reporting. For further information on Nature Research policies, see our Editorial Policies and the Editorial Policy Checklist.

Statistics

For all statistical analyses, confirm that the following items are present in the figure legend, table legend, main text, or Methods section.

n/a	Confirmed	
☐	☒	The exact sample size (*n*) for each experimental group/condition, given as a discrete number and unit of measurement
☐	☒	A statement on whether measurements were taken from distinct samples or whether the same sample was measured repeatedly
☐	☒	The statistical test(s) used AND whether they are one- or two-sided<br>*Only common tests should be described solely by name; describe more complex techniques in the Methods section.*
☒	☐	A description of all covariates tested
☐	☒	A description of any assumptions or corrections, such as tests of normality and adjustment for multiple comparisons
☐	☒	A full description of the statistical parameters including central tendency (e.g. means) or other basic estimates (e.g. regression coefficient) AND variation (e.g. standard deviation) or associated estimates of uncertainty (e.g. confidence intervals)
☐	☒	For null hypothesis testing, the test statistic (e.g. *F*, *t*, *r*) with confidence intervals, effect sizes, degrees of freedom and *P* value noted<br>*Give P values as exact values whenever suitable.*
☒	☐	For Bayesian analysis, information on the choice of priors and Markov chain Monte Carlo settings
☐	☒	For hierarchical and complex designs, identification of the appropriate level for tests and full reporting of outcomes
☒	☐	Estimates of effect sizes (e.g. Cohen's *d*, Pearson's *r*), indicating how they were calculated

Our web collection on statistics for biologists contains articles on many of the points above.

Software and code

Policy information about availability of computer code

Data collection	Flow cytometry data were collected on LSRFortessa (BD) instrument with FACSDiva software v8.0.1 (BD)
Data analysis	Flow cytometry data were analyzed on FlowJo v10.4 (TreeStar) and statistical analysis performed on Prism v8.0.1 (GraphPad).<br>Genomic analyses were performed using R Studio 3.6.3 and all packages utilized were updated to most current versions. Genomic data was visualized on IGV 2.8.3 version.

For manuscripts utilizing custom algorithms or software that are central to the research but not yet described in published literature, software must be made available to editors and reviewers. We strongly encourage code deposition in a community repository (e.g. GitHub). See the Nature Research guidelines for submitting code & software for further information.

Data

Policy information about availability of data

All manuscripts must include a data availability statement. This statement should provide the following information, where applicable:

- Accession codes, unique identifiers, or web links for publicly available datasets
- A list of figures that have associated raw data
- A description of any restrictions on data availability

The scRNAseq and ATAC-seq data is available at GEO under accession number GSE198423(https://www.ncbi.nlm.nih.gov/geo/query/acc.cgi?acc=GSE198423). The mouse reference genome mm10 was used for scRNAseq and ATAC-Seq analysis.
Other datasets were used in this study: Carmona et.al, 2020 (doi: 10.1080/2162402X.2020.1737369), Bengsch et.al, 2018 (doi: 10.1016/j.immuni.2018.04.026). All the data supporting the findings of this study are available within the article, the Supplementary Information and Source Data file. All other relevant data are available from the corresponding author on request.

Field-specific reporting

Please select the one below that is the best fit for your research. If you are not sure, read the appropriate sections before making your selection.

☒ Life sciences ☐ Behavioural & social sciences ☐ Ecological, evolutionary & environmental sciences

For a reference copy of the document with all sections, see nature.com/documents/nr-reporting-summary-flat.pdf

Life sciences study design

All studies must disclose on these points even when the disclosure is negative.

Sample size	No statistical method was used to predetermine sample size. Sample sizes were based on those used in previous and preliminary studies from our lab which allow for statically valid comparisons. For single cell RNA-seq experiments, more than 30000 cells were collected from each sample, 2 replicates were used for each group.. For ATAC-seq experiment, 50000 cells were collected from each sample, 2 replicates were used for each group.
Data exclusions	No data were excluded from analyses.
Replication	All data were reliably reproduced in at least two independent experiments. All presented results were repeatable.
Randomization	For in vivo tumor growth experiments, mice were randomized prior treatment. Age and sex-matched animals were used for each experiment. WT and KO mice were littermates if possible.
Blinding	Blinding was not achieved due to requirements for cage identification and labeling for treatment pourpouses.

Reporting for specific materials, systems and methods

We require information from authors about some types of materials, experimental systems and methods used in many studies. Here, indicate whether each material, system or method listed is relevant to your study. If you are not sure if a list item applies to your research, read the appropriate section before selecting a response.

Materials & experimental systems

n/a	Involved in the study
☐	☒ Antibodies
☐	☒ Eukaryotic cell lines
☒	☐ Palaeontology and archaeology
☐	☒ Animals and other organisms
☒	☐ Human research participants
☒	☐ Clinical data
☒	☐ Dual use research of concern

Methods

n/a	Involved in the study
☒	☐ ChIP-seq
☐	☒ Flow cytometry
☒	☐ MRI-based neuroimaging

Antibodies

Antibodies used	Annexin APC BLE640930 OZYME; CD16/CD32 (Mouse BD Fc Block™) Clone 2.4G2 (RUO) 553142 BD.<br>Primary antibodies listed as antigen first, followed by clone, catalog number, supplier and dilution.<br>BCL-2, BCL/10C4, 633508, Biolegend, PE, 1:100; CD4, RM4-5, 564933, BD, BUV737, 1:200; CD4, GK1.5, 563790, BD, BUV395, 1:200; CD8a, 53-6.7, 563786, BD, BUV395, 1:200; CD8a, 53-6.7, 564297, BD, BUV737, 1:200; CD8a, 53-6.7, 562315, BD, PE-CF594, 1:1600; CD11b, M1/70, 101261, Biolegend, APC-Cy7, 1:3200; CD11b, M1/70, 101216, Biolegend, PE-Cy7, 1:1000; CD11c, N418, MCD11C17, Invitrogen, PE-Texas Red, 1:200; CD19, 6D5, 115520, Biolegend, PE-Cy7, 1:1600; CD26, H194-112, 137806, Biolegend, FITC, 1:100; CD38, 90/CD38, 562770, BD, PerCP-Cy™5.5, 1:200; CD39, 24DMS1, 25-0391-82, Invitrogen, PE-Cy7, 1:100; CD44, IM7, 103047, Biolegend, BV605, 1:800; CD44, IM7, 103049, Biolegend, BV650, 1:400; CD45.2, 104, 109822, Biolegend, Alexa Fluor 700, 1:1600; CD45.2, 104, 564880, BD, BUV737, 1:100; CD62L, MEL-14, 553152, BD, APC, 1:1000; CD62L, MEL-14, 740660, BD, BV711, 1:1600; CD62L, MEL-14, 533150, BD, FITC, 1:1600; CD64, X54-5/7.1, 139306, Biolegend, APC, 1:100; CD101, 307707, 564473, BD, Alexa Fluor 647, 1:100; CD172a (SIRP alpha), P84, 46-1721-82, Invitrogen, PerCP-eFluor710, 1:100; CD223 (LAG-3), C9B7W, 125221, Biolegend, BV421, 1:100; CD244.2 (2B4), eBio244F4, 11-2441-82, eBioscience, FITC, 1:100; CD279 (PD-1), 29F.1A12, 135225, Biolegend, BV785, 1:100; CD335 (NKp46), 29A1.4, 25-3351-82, eBioscience, PE-Cy7, 1:200; CD366 (Tim-3), RTM3-23, 119706, Biolegend, APC, 1:100; CD366 (Tim-3), RTM3-23, 134004, Biolegend, PE, 1:100; EOMES, Dan11mag, 61-4875-80, eBioscience, PE-eFluor 610, 1:100; EOMES, Dan11mag, 46-4875-82, eBioscience, PerCP-eFluor 710, 1:200 ; F4/80, BM8, 123141, Biolegend, BV785, 1:100; F4/80, BM8, 25-4801-82, eBioscience, PE-Cy7, 1:100; Granzyme B, GB11, 515408, Biolegend, Pacific Blue, 1:100; H-2K[b], AF6-88.5, 562832, BD, Alexa Fluor 647, 1:50; ISG15, F-9, sc-166755, Sta Cruz Bio., PE, 1:200; IFNg, B27, 564039, BD, BV711, 1:100; Ki67, B56, 556027, BD, PE, 1:80; MHC Class II (I-A/I-E), M5/114.15.2, 48-5321-82, Invitrogen, eFluor 450, 1:1000; NK1.1, PK136, 108716, Biolegend, PE-Cy5, 1:400; NK1.1, PK136, 552878, BD, PE-Cy7, 1:200; SLAMF6 (Ly-108), 13G3, 740090, BD, BV421, 1:100; Tbet, 4B10, 644824, Biolegend, PE-Cy7, 1:400; TCF1, C63D9, 6444S, Biolegend, Alexa Fluor 488, 1:100; TCRb, H57-597, 109220, Biolegend, APC-Cy7, 1:400; TCRb,

	560729, 566345, BD, PE-Cy7, 1:200; TOX, TXRX10, 12-6502-82, Invitrogen, PE, 1:100; XCR1, ZET, 148220, Biolegend, BV650, 1:200.
Validation	Representative flow panels are shown in Supplementary Figure 7. Further validation is present on the manufacturer's website. Antibodies were all titrated to determine the optimal concentration. Titration stainings included additional markers in order to validate the expression patterns on know subsets (e.g. T cells). Optimal concentration was defined by comparing the expression with other validated clones of a given antibody or with other previously validated lots of the same antibody clone.

Eukaryotic cell lines

Policy information about cell lines

Cell line source(s)	mastocytoma cells P815-GFP derived from DBA/2 (H-2d) mice (gift from Dr. B. Salomon - Hopital Pitié Salpétrière, Paris-France)<br>melanoma cells B16F10 (B16) and melanoma cells B16F10 expressing OVA (B16-OVA) (gift from Dr. K. L. Rock - Dana Farber Cancer Institute, Boston, USA)<br>lymphoma cells EL4 expressing OVA (EL4-OVA)<br>fibrosarcoma cells (MCA-101) (gift from Dr. L. Zitvogel - IGR, Villejuif, France)
Authentication	None of the cell lines used were authenticated.
Mycoplasma contamination	All cell lines were tested for mycoplasma contamination prior to use in experiments.
Commonly misidentified lines<br>(See ICLAC register)	No commonly misidentified cell lines were used.

Animals and other organisms

Policy information about studies involving animals; ARRIVE guidelines recommended for reporting animal research

Laboratory animals	C57BL/6J (JAX #000664)<br>B6D2F1 (JAX#100006)<br>OT-I (JAX#003831)<br>B6.SJL-Ptprca Pepcb/BoyJ (CD45.1) (JAX #002014)<br>C57BL/6J males (H-2b) and C57BL/6J female hybrids B6D2F1 [B6xDBA/]2 F1 (H-2bxd) mice were obtained from Charles River Laboratories and used at 8-10 weeks of age. Suv39h1tm1Jnw C57BL/6J (Suv39h1-KO) mice were provided by T. Jenuwein, were backcrossed with C57Bl/6J mice for at least nine generations. C57BL/6J-Tg(TcraTcrb)1100Mjb/J mice (OT-I), and B6.SJL-Ptprca Pepcb/BoyJ (CD45.1) mice were purchased from Jackson lab. OT-I mice were crossed with CD45.1 mice to obtain OT-I/CD45.1 mice. Suv39h1Flox/Flox (official name is B6-Suv39h1tm1Ciphe) were originally produced at the Centre d'Immunologie de Marseille (CIPHE, B. Malissen) and were subsequently bred in the animal facility of Institut Curie. The strategy targets the exon 2 from the transcript Suv39h1-001 ENSMUST00000115638. LoxP sites were introduced in the intron 1 at 70pb in 5'of exon 2 and in the intron 2 at 98 pb in 3' of exon 2. The loxP site in the intron found at the 3' end of exon 2 was abutted to a Frt-neoR-Frt cassette. After deletion of the Frt-neoR-Frt cassette by flipage, a residual Frt site will remain just after the loxP 3'. CD4-Cre transgenic mice were obtained from Jacques Ghysdaël (Institut Curie). Suv39h1-Flox*CD4-Cre+/- and control Suv39h1-Flox*CD4-Cre-/- mice were used for the experiment.
Wild animals	Study did not involve wild animals.
Field-collected samples	Study did not involve samples collected in the field.
Ethics oversight	All animal procedures were in accordance with the guidelines and regulations of the Institut Curie veterinary department. Animal care and use for this study were performed in accordance with the recommendations of the European Community (2010/63/UE) for the care and use of laboratory animals. Experimental procedures were specifically approved by the ethics committee from Institut Curie, officially registered as CEEA-IC #118 and the Ministère de l'enseignement supérieur, de la recherche et de l'innovation which validated the project with the reference (APAFIS#12325_20171124123634-v2) in compliance with the international guidelines. Mice used in the experiments were age- sex- matched and were euthanized by cervical dislocation. Mice breeding were in SPF animal facilities and experimental and control animals were co-housed with housing conditions using a 12 light/12 dark cycle, with a temperature between 20-24°C with an average humidity rate between 40-70%.<br>Humane endpoints are used for mice bearing tumors, ie. the maximal ethical size of tumors subcutaneously grafted is 2000mm3, or more than 20% of weight loss or any signs of altered mobility or eating ability, or cachexia.

Note that full information on the approval of the study protocol must also be provided in the manuscript.

Flow Cytometry

Plots

Confirm that:

☒ The axis labels state the marker and fluorochrome used (e.g. CD4-FITC).

☒ The axis scales are clearly visible. Include numbers along axes only for bottom left plot of group (a 'group' is an analysis of identical markers).

☒ All plots are contour plots with outliers or pseudocolor plots.

☒ A numerical value for number of cells or percentage (with statistics) is provided.

Methodology

Sample preparation	Blood cells were harvested on day 12 post tumor implantation while DLN, spleen and tumor were harvested at day 19-20 post tumor implantation by mechanical disruption. Red blood cells were lysed with hypotonic buffer, single-cell suspensions were prepared in PBS 0.5% BSA and 2 mM EDTA (FACS buffer). Inguinal DLN and spleen were collected in CO2 Independent medium (GIBCO). Single-cell suspensions were obtained by mechanical disruption over a 40um cell strainer. Tumors were digested in 2 ml of CO2 Independent medium containing 0,1mg/ml DNase I and 0,1mg/ml Liberase TL (Roche) at 37°C for 30 min in agitation. Samples were transferred to C tubes (Miltenyi Biotec) for mechanical dissociation with GentleMACS and cell suspension was then filtered with a 100um cell strainer. Mononuclear cells were recovered from Percoll gradient (GE Healthcare Life Science) from 40% to 75% interface, washed and resuspended in FACS buffer. Isolated cells were stained with antibodies and/or tetramers, as described in Methods sections.
Instrument	BD LSR Fortessa was used to collect data for analysis. BD FACSAria III was used for cell sorting.
Software	All flow data was collected using LSRFortessa (BD) instrument with FACSDIVA 8.0.1 (BD Pharmingen) and analyzed using FlowJo version 10.4.1 (TreeStar).
Cell population abundance	All sorts had a purity > 95%, checked by post-sort re-sampling.
Gating strategy	Initial general gating strategy: FSC_A/SSC_A was used to gate on lymphocytes. Doublets were excluded through FSC-A/FSC-H. Dead cells positive for LIVE/DEAD fixable AQUA were excluded. CD45 cells were gated as CD45.2 positive and FSC-A. TCRb cells were gated as positive and FSC-A. CD8 T cells were gated as CD8a positive and CD4 negative. These were followed by gating strategy described in the figures and figure legends.<br>General gating strategy for sorting: FSC_A/SSC_A and FSC_A/FSC_W was used to gate on lymphocytes. Doublets were excluded through SSC-A/SSC-W. Dead cells positive for DAPI and lineage (CD19, NK1.1, F4/80) were excluded. CD45 cells were gated as CD45.2 positive and CD8/TCRb double positive cells were gated to be sorted.<br>FMO controls were used to determine negative and positive populations.

☒ Tick this box to confirm that a figure exemplifying the gating strategy is provided in the Supplementary Information.
